# Fault detection and diagnosis of the wastewater nitrate and nitrite sensors using PCA and FDA combined with assessment of the economic and environmental impact of the faults

**DOI:** 10.1007/s10661-024-13593-z

**Published:** 2025-01-02

**Authors:** Alexandra-Veronica Luca, Melinda Simon-Várhelyi, Norbert-Botond Mihály, Vasile-Mircea Cristea

**Affiliations:** Department of Chemical Engineering, Faculty of Chemistry and Chemical Engineering, Babes-Bolyai University of Cluj-Napoca, 11 Arany János Street, 400028 Cluj-Napoca, Romania

**Keywords:** Energy costs evaluation, Fault detection, Fault identification, Greenhouse gasses emissions, Nitrate and nitrite sensor

## Abstract

One of the leading challenges in Water Resource Recovery Facility monitoring and control is the poor data quality and sensor consistency due to the tough and complex circumstances of the process operation. This paper presents a new principal component analysis fault detection approach for the nitrate and nitrite concentration sensor based on Water Resource Recovery Facility measurements, together with the Fisher Discriminant Analysis identification of fault types. Five malfunction cases were considered: constant additive error, ramp changing error in time, incorrect amplification error, random additive error, and unchanging sensor value error. The faults’ implementation, fault detection, and identification methods are presented and evaluated in terms of accuracy and promptitude. The models are originating from a municipal plant. The amount of required electrical energy and greenhouse gas released during the Water Resource Recovery Facility operation were assessed for the cases of nitrates and nitrites NO sensor normal and malfunctioning regimes. The environmental and economic evaluations show the benefits of detecting and identifying nitrates and nitrites NO sensor defects aimed at providing efficient and environmentally friendly operation of the Water Resource Recovery Facility. The fault-affected operation cases showed increased values, up to 10% for the total energy demand and 4% for the total greenhouse gas emissions, when they are compared to the normal operation case.

**Supplementary Information:**

The online version contains supplementary material available at 10.1007/s10661-024-13593-z.

## Introduction

Mathematical modelling has become a worthful tool for finding potential improvements to be achieved by advanced control strategies of the Water Resource Recovery Facility (WRRF) (Makinia & Zaborowska, 2010; Nair et al., 2018). The family of activated sludge models (ASMs) No. 1, 2, 2d and 3 (Henze et al., 2000) and the benchmark simulation models (BSMs) No. 1 (Alex et al., 2008) and 2 (Jeppsson et al., 2007) succeed to provide a first principle-based explanation of the WRRF processes. The ASMs define the frame of the biochemical transformations, while the BSMs implement them in benchmark WRRF configurations of typical design, capacity and operation, including complementary units to the bioreactors. The BSM1 may also comprise two control loops, one for the dissolved oxygen (DO) control in aerobic reactors and another one for the nitrate (${\text{NO}}_{3}^{-})$ and nitrite $({\text{NO}}_{2}^{-})$ concentration control in the anoxic reactors (Haimi et al., 2010). The first guarantees efficient nitrification, while the later assures appropriate denitrification performed by bacteria. Both of them are important for water quality but the denitrification step is crucial since nitrate and nitrite (NO) can be harmful for human health (Parvizishad et al., 2017), toxic to aquatic life (Cristea, 2013), can lead to chlorination problems (Curtin et al., 2011) and eutrophication (Soares et al., 2011).

WRRF operation has to deal with multiple constrains, included in dedicated regulations. Some of them are imposed at national levels. Others, such as the European Directive 91/271/EEC of 1991, are set for community of countries. These requirements emerge in the objective of efficient process operation, attainable by the implementation control strategies (Olsson et al., 2005). There are many studies on effluent quality (Santin et al., 2016; Ma et al., 2019), reduced energy consumption (Di Fraia et al., 2018; Simon-Varhelyi et al., 2020) and WRR processes optimization (Borzooei et al., 2019; Zhou et al., 2021). Sensors represent a prerequisite for achieving these objectives in real-time performance monitoring (Corona et al., 2013).

Despite the current trend aims to achieve energy neutrality (IWA, 2018) and leads the process to the most efficient reduction of emissions, this has only been accomplished in rare real-life WRRFs (EUDP, 2017). Recent studies on WRRF GHGs emissions used fault tree analysis as potential failure detector (Bourouni, 2013; Ba-Alawi et al., 2020). Different works have addressed indirect emissions related to energy consumption (Parravicini et al., 2016; Marinelli et al., 2021) and direct WRRF emissions (Campos et al., 2016; Parravicini et al., 2022). Other studies have focused on diverse control strategies to reduce the emissions (Santin et al., 2017; Qandil, et al., 2021) or the energy demand (Budych-Gorzna et al., 2021). Several publications investigated economic evaluation of GHG emissions and electrical consumption (Gemar et al., 2018; Yapicioglu & Yesilnacar, 2022; Jimenez-Benitez et al., 2023), but none addressed the topic of combined environmental and economic impact of faulty sensors.

Intelligent sensors proved to be valuable for obtaining quantitative and qualitative assessments of variables when traditional measurements are not available, expensive or unreliable (Haimi et al., 2015). Soft-sensors aim to solve two other major problems: recognition and isolation of hardware sensor faults (Haimi et al., 2013; Yan et al., 2020). Faulty operation of concentration sensors of WRRFs can significantly reduce control system setpoint tracking performance, affect its disturbance rejection potential, raise operational costs and lead to poor quality of the effluent. Inaccurate nitrogen compounds monitoring and treatment can lead to elevated on-site and off-site CO_2_ and N_2_O emissions, along with a decline in satisfying environmental standards and overall treatment effectiveness. Taking this into consideration, fault detection is crucial for maintaining optimal process operation. Early failure detection helps avoid plant inefficiency, increased costs and breakdowns. Process monitoring ensures accurate plant performance by achieving four distinct tasks: (1) fault detection reveals if an aspect in the process needs correction; (2) fault diagnosis or error identification reveals the source of the issue; (3) fault estimation evaluates the fault magnitude; and (4) fault reconstruction computes the fault-free process variable values aimed at performing reliable process operation in the presence of defects (Qin, 2003).

Multivariate statistical process control (MSPC) methods proved to be efficient in fault detection (Qin, 2009; Corominas et al., 2011). Among them are the principal component analysis (PCA) (Tao et al., 2013; Dong & Qin, 2018; Luca et al., 2021), partial least squares (PLS) (Chen et al., 2016, Liu et al., 2021), independent component analysis (ICA) (Villegas et al., 2010; Yu, 2012) and support vector machine (SVM) (Kazemi et al., 2021). MSPC is ideal in fault detection because it relates on the latent variables. In contrast, simple statistical process control tools, as are control charts, directly use measured variables (Garcia-Alvarez, 2009).

Data-driven models are based on the measured data, they describe the factual behaviour, providing a good description of the process conditions (Haimi et al., 2015; Mamandipoor et al., 2020) but do not necessitate a detailed explanation of the intrinsic phenomena, mechanisms or fundamental system knowledge (Ma et al., 2021). Data-driven fault detection unveils the existence of a faulty situation (Schneider et al., 2019), especially in complex, large-scale, ill-defined, non-linear and time-varying systems (Li & Yan, 2019). Fault detection algorithms are not easily applicable to wastewater systems due to the uncertainty of multiple combinations of complex factors (Liu et al., 2023). The WRRF advanced or plantwide control is even more difficult to achieve because a large plant may involve up to 30,000 measured signals (Olsson et al., 2014). The most frequently used data-driven MSPC methods is PCA because it offers an essential representation of process variables (Amin et al., 2018). Additionally, PCA is suitable for detecting sensor faults by reducing dimensionality and enhancing interpretability while minimizing information loss. It achieves this by generating new uncorrelated variables that maximize variance and reflect hidden correlations between variables.

When considering a large number of studies considering out-of-range values of the WRRF NO concentration signal (Rieger et al., 2008; Britschgi et al., 2020; Juncal et al., 2020), only few inspected the fault detection and diagnosis. Some used the ICA (Yu, 2012) or the PCA methods to detect the malfunctions. One of the PCA focused papers developed a structured residual approach with maximum sensitivity method to detect sensor faults, such as bias, drift, complete failure and precision degradation (Yoo et al., 2006). Another showed that soft-sensor values estimation is preferred when hardware instrumentation measurements are faulty. In this case a reflection-based PCA algorithm was used (Haimi et al., 2015). A different study proposed the hybrid methodology built on PCA with T^2^ statistics and the Bayesian network (BN) (Amin et al., 2018).

FDA is a popular pattern classification technique (He et al., 2005; Fuente et al., 2008). The application of FDA to malfunctioning sensors in air handling systems was thoroughly investigated (Du & Jin, 2008). FDA was also investigated for the diagnosis of DO sensors (Luca et al., 2023). FDA delivers the optimal lower dimensionality illustration for fault identification on the basis of a distinguishing factor between categories of data, upon which every class represents data gathered over a definite and known error type. In contrast with PCA, that seeks directions which are effective in terms of representation, FDA seeks directions that are effective in terms of discriminatory information in sensor data. As a result, FDA has theoretical benefits for fault detection and diagnosis (Chiang et al., 2000).

This work focuses on the detection and identification of five faults that affect the NO concentration sensor, as this is the main component of the ${\text{NO}}_{3}^{-}$ and ${\text{NO}}_{2}^{-}$ control loop typically present in activated sludge processes. The implemented faults are: constant additive error, ramp changing error in time, incorrect amplification error, random additive error and unchanging sensor value. Considering the advantages of the PCA and FDA for the detection and diagnosis of sensors faults, the present paper proposes the PCA model for fault detection, along with the FDA model for fault identification of defective functioning scenarios associated to NO concentration sensor in the dynamic operation of WRRF. The models are based on normal operation sets of data obtained and reconciled from a Romanian WRRF. The dynamic simulations for normal and sensor fault-affected process were built using Simulink™, the graphical extension of MATLAB. The novelty of this work consists in its comprehensive approaches to detect and identify a specific set of faults affecting the NO concentration sensor belonging to the NO control loop, working in association with the DO control loop of a municipal WRRF. The work innovatively combines PCA for fault detection and FDA for fault identification, aiming the sustainable and automated WRRF operation. Additionally, the study evaluates the fault detection and identification capabilities of the model using precision, recall, accuracy, MAR (Missed Alarm Rate) and the F1 score along with the economic and environmental impacts of sensor faults, providing insights for optimizing WRRF performance. The electricity expenses and environmental effects on WRRF performance were evaluated using specific metrics for the urban WRRF case under study and for each of the NO sensor failure types.

The paper is organized to present (i) the WRRF model employed for dynamic simulations, (ii) the theoretical bases of the PCA fault detection and FDA fault diagnosis approach for the five different types of investigated NO sensor errors, (iii) the overview of the metrics employed to calculate the amount of GHG released by the sensor induced faulty process, and (iv) the findings of the WRRF performance gained by analysing the sensor faults affected operation, the economic and environmental assessments, ending the study with (v) the conclusions of the investigations for enhancing the operation of the WRRF considered as case study.

## Methods

### Municipal WRRF and process model

The Romanian municipal WRRF considered as case study is located in Someseni, Cluj County. The layout of the plant is an anaerobic-anoxic–oxic (A^2^O) one, according to the design and operation of the bioreactors, working in association with the primary and secondary clarifiers.

**Fig. 1:**
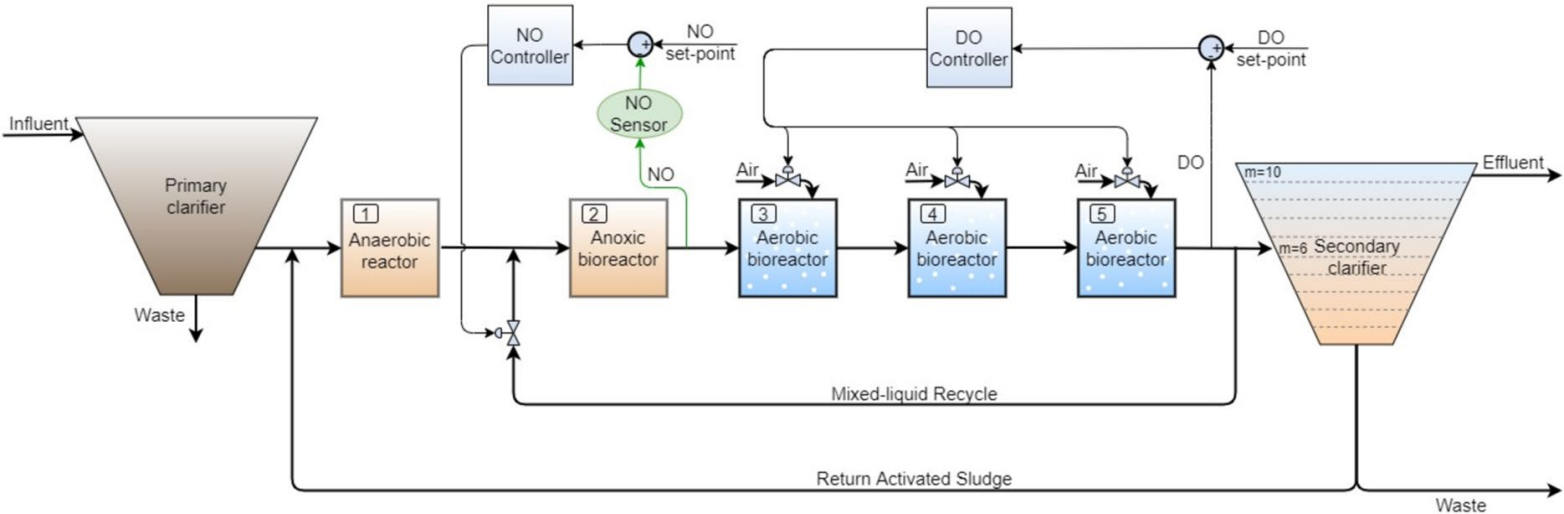
Layout of the municipal WRRF and its main DO and NO control loops

The layout representation of the investigated municipal WRRF is shown in Fig. 1.

In the primary treatment step, upstream from the primary clarifier, the large objects, sand and grease are first removed. After this separation, downstream from the first settler, the wastewater reaches the anaerobic reactor where phosphate-accumulating bacteria sustain the process of phosphorous removal. In the anoxic reactor heterotrophic bacteria facilitate denitrification (Cappuccino & Sherman, 2014). The biochemical transformations in the anoxic reactor enable the nitrites and nitrates reduction, leading to nitrous oxide and dinitrogen gas release (Wanner & Grau, 1988). In the aerobic reactors of the nitrification stage, influent ammonia (NH_3_) is transformed into nitrites and nitrates by autotrophic aerobic bacteria (Gerardi, 2002). The formed nitrites and nitrates (NO) are returned to the anoxic basin for denitrification by the mixed-liquid recycle stream. The gravitation-based separation between sludge and treated water occurs in the secondary clarifier. The clean effluent is discharged into the receiving river and the activated sludge waste leaving the secondary settler is sent to the anaerobic digestion unit to be used for biogas production. The rest is returned to the anaerobic zone to sustain biomass development by the return activated sludge stream. Details on the municipal WRRF equipment design parameters are presented in Table S1 of the Supplementary Information (SI). The plant serves approximately 414,000 inhabitants and has a maximum influent flow rate of about 150,000 m^3^/day. The average values of influent, effluent components and operating variables can be found in Table S2 of the Supplementary Information (SI).

The dynamic simulator of the A^2^O municipal WRRF was built using the mathematical models of the Otterpohl primary settler (Otterpohl & Freund, 1992), the biological reactors described according to ASM1 (Henze et al., 2000; Rieger et al., 2012) and the Takacs secondary settler model (Takacs et al., 1991). Although ASM1 does not explicitly model phosphorus removal, the anaerobic zone was simulated without the presence of dissolved oxygen, nitrates or nitrites, capturing the essential biological processes within the framework of ASM1. The anoxic and aerobic zones were directly modelled using ASM1, with a focus on nitrogen removal processes. The calibration of the WRRF dynamic simulator was performed in a previous work (Varhelyi et al., 2019) using both design, operational and measured data, collected from the municipal Someseni WRRF. This confirmed that ASM1 could reliably represent the carbon and nitrogen dynamics of the plant, adapted for its A^2^O configuration. Details on the WRRF model calibration approach based on different optimization approaches and results of the model fit to the measured data are presented in (Varhelyi et al., 2019) and (Nair et al., 2018).

The matrix of operation data used for the construction of the PCA model was collected from the previously calibrated model of the municipal WRRF. The influent of the WRRF corresponds to dry weather conditions. This ASM1 model, implemented for the A^2^O plant layout, considers the oxidation of influent carbonaceous biological matter, in association with the nitrification and denitrification processes for ammonia removal. The WRRF performance indices and control strategies proposed in BSM1 were used for the evaluation of the effluent quality, pumping and aeration energy, while a set of additional equations were used for assessing the GHG emissions.

The control system implemented in the dynamic simulator regulates the nitrification and denitrification processes and has the typical configuration for large and continuously operating WRRFs. Specifically, two feedback control loops with proportional-integral (PI) controllers were implemented, as also shown in Fig. 1. The first one includes a dissolved oxygen controller aimed to keep the DO concentration in the fifth reactor to the set-point value of 2 mg O_2_/L. The manipulated variable of this DO control loop is the air flow rate supplied to the aerated bioreactors. The nitrates and nitrites concentration in the anoxic reactor is the controlled variable of the second (NO) control loop. Its set-point value is 0.01 mg N/L. Despite the challenges associated with controlling both parameters, the effectiveness of this control strategy is well-supported by the literature, demonstrating its capability to maintain robust control performance (Aparna et al., 2024; Sheik et al., 2021; Tejaswini et al., 2020). The mixed-liquid recycle flowrate is the manipulated variable for this control loop.

### Sensors faults and their implementation

The types of the NO sensor faults considered in the present study are constant additive error (bias), ramp changing error in time (drift), incorrect amplification (wrong gain), random additive error (loss of accuracy) and unchanging sensor value (fixed value).

These sensor faults were implemented in the WRRF simulator following the following scenario. The first 139 days of the simulation consisted in a fault-free operation. Then, starting from the 140^th^ day of the simulation, each of the type faults was individually introduced and maintained for the next 20 days. Data containing the effect of each faulty sensor operation on the WRRF variables were collected for this last period of time and used to perform the PCA fault detection and FDA fault diagnosis. The mathematical description of the fault types is presented in Table S3 of the Supplementary Information.

Constant additive error is caused by the miscalibration of the sensor. It is also known as the shift or bias fault because the delivered value is shifted with a constant value, in comparison with the true one (Teh et al., 2020). The fault was implemented in the simulation by summing up a negative constant value of − 0.0098 mg N/L to the true NO concentration value.

The time continuously (decreasing) deviation from true value of the NO faulty variable is called ramp changing error in time. It is characterized by a ramp error signal (Teh et al. 2020). The fault was implemented by integrating in time the constant value of − 0.0075 mg N/L and adding the integrated signal to the actual NO process value.

When the sensor’s effectiveness deteriorates, a gain calibration failure occurs. This fault, called incorrect amplification, appears as a poor correlation between the input and output variables when the sensor’s calibration slope is inexactly set during its calibration process (Rosen et al., 2008). A wrong gain of 0.3 was considered in the study. To gradually introduce in time the sensor’s incorrect gain of 0.3 mg N/L, a first order filter with a time constant of 0.1 days was used for simulating this fault.

Random additive error is an irregular degradation of the sensor performance. Because the value of the sensor’s signal is randomly fluctuating around the true value, this fault type is difficult to identify (Teh et al., 2020). An additional value that fluctuates between − 0.06 mg N/L and + 0.06 mg N/L was randomly generated and added to the true NO process value. The output signal of the faulty sensor was limited to a minimum positive value of 10–15 mg N/L. Each of the random added value lasted for a period of time of 0.1 day.

Unchanging sensor value type of fault was simulated by locking the sensor signal to a constant value (Rosen et al., 2008). In the present study, the constant value of 0.01 mg N/L was chosen.

The calibrated simulator for the municipal WRRF and the associated software modules for including the faults were implemented in SimulinkTM software environment. All S-functions in the dynamic simulator were written as C-files and compiled to executable forms in order to considerably reduce the simulation time.

### Principal component analysis algorithm for fault detection

PCA is a data-mining methodology of dimensionality-reduction based on a process model constructed with conventional process originated data. PCA converts a large set of variables into a smaller one that captures the relationship between them. Using PCA, the original data space is divided in two subspaces—a principal component subspace (PCS) and a residual subspace (RS). Where the measurements number is higher than the states number, PCA simplifies monitoring and fault detection (Wise et al., 1990). PCA is often used with Hotelling’s T^2^ and square prediction error (SPE) metrics. These are described in SI. The multivariate Hotelling’s T^2^ is the correspondence of the univariate *t*-test and offers a complete measure of the variations in PCS (Jackson, 2003). The T^2^ method is generally used to identify fluctuations in the operating environment (Yoo et al., 2004). SPE measures the variations in the RS as a sum of squares of residuals. A higher value of ${T}_{\alpha }^{2}$ or $SPE$ than the corresponding threshold indicates an abnormal operation.

PCA model construction requires choosing the correct number of principal components to describe the system. If fewer principal components are chosen than required, an insufficient representation of the process will be obtained. Otherwise, the model will be overparametrized. The cumulative percent variance (CPV) offers reliability for finding the representative principal components number (Valle et al., 1999).

In the present study, the standard PCA was used as it was found to provide good detection results, reducing complexity and sparing computation resources implied by the dynamic PCA fault detection approach for the real-time application.

### Fisher discriminant analysis algorithm

Fisher discriminant analysis (FDA) is a pattern classification method with a high categorization potential. FDA seeks the discriminant vector that performs the greatest separation between classes in the projected data space. Fisher’s linear discriminant maximizes the distance between projected means of classes and minimizes variance projected within-class. The FDA algorithm is thoroughly presented in SI, associated with a summarized flow diagram.

### Assessment metrics for fault detection and identification

The confusion matrix was computed and utilized for each individual case to rigorously assess the performance of the fault detection and identification methods across both normal and abnormal conditions. This approach enabled the calculation of various performance metrics, including precision, recall, accuracy, FDR and MAR. Additionally, the F1 score was employed to offer a balanced assessment by integrating precision and recall into a single metric. These values were derived directly from the confusion matrix, allowing for an in-depth analysis of the system’s proficiency in accurately identifying defects and minimizing false detections.

The rows of the confusion matrix represent the predicted classes (Output Class), while the columns represent the actual classes (Target Class). Correctly classified observations are located in the diagonal cells, whereas misclassified observations appear in the off-diagonal cells. Each cell displays both the count of observations and the corresponding percentage of the total observations. The rightmost column of the matrix indicates the percentage of instances predicted for each class that were classified correctly or incorrectly, corresponding to metrics known as precision (or positive predicted value) and the false discovery rate or FDR, respectively. Similarly, the bottom row shows the percentage of actual instances of each class that were classified correctly or incorrectly, corresponding to recall (or true positive rate) and the false negative rate or MAR, respectively. The overall accuracy of the classification is presented in the bottom-right cell of the matrix. The calculations and subsequent analysis were carried out using MATLAB, which facilitated data processing and evaluation through its built-in statistical analysis functions.

### Assessment of the GHG impact

WRRFs contribute to GHGs release due to the inherent energy demand and gaseous emissions from wastewater processing. This paper highlights the variations in plant performance, in terms of environmental impact, effluent quality and energy costs, when it is operated regularly, versus the improper operation due to the faulty NO sensor. General impact assessment on dysfunctional plant operation performance was conducted using the following performance indices: aeration energy (AE), pumping energy (PE) and effluent quality (EQ) in association to the GHG emissions assessment. As indicated in Eq. (1), the AE index is calculated using the oxygen mass transfer coefficient of aerobic bioreactors (*K*_*L*_*a*), which is directly related to the air flowrate:

1 $$AE=\frac{{SO}_{sat}}{T\cdot 1.8\cdot 1000}\cdot \underset{0}{\overset{T}{\int }}\sum \limits_{aerated\;reactors}{V}_{bioreactor}\cdot {K}_{L}{a}_{i}(t)dt$$

where the used parameters are explained in Alex et al., 2008.

The pumping energy index is determined by three different flow rates:

2 $$PE=\frac{1}{T}\cdot \underset{0}{\overset{T}{\int }}\left[0.004\cdot {Q}_{NR}\left(t\right)+0.08\cdot {Q}_{RAS}\left(t\right)+0.05\cdot {Q}_{waste}(t)\right]dt$$

where thorough explanations of the utilized parameters can be consulted in Alex et al. (2008).

Total suspended solids (TSS), chemical oxygen demand (COD), biochemical oxygen demand (BOD), total Kjeldahl nitrogen (TKN) and NO concentrations in the effluent flow stream are used to calculate the EQ, as shown in Eq. (3). This index is measured in kilograms of pollutant units/day:

3 $$EQ=\frac{1}{T\cdot 1000}\cdot \underset{0}{\overset{T}{\int }}\left[{PU}_{TSS}\left(t\right)+{PU}_{COD}\left(t\right)+{PU}_{BOD}(t)+{PU}_{TKN}\left(t\right)+{PU}_{NO}(t)\right]\cdot \ {Q}_{effluent}\left(t\right)dt$$

where detailed explanations of the used parameters can be consulted in Alex et al. (2008).

GHGs from the first step of water processing include on-site and off-site emissions of CO_2_ and N_2_O gases. Aside from CO_2_, N_2_O is regarded as a substantial contributor to GHGs due to its global warming potential (GWP) of approximately 265–298 times higher factor than the CO_2_ gas, considering a mean residence time of 100 years (Vallero, 2019).

Off-site CO_2_ emissions (kg CO_2_/day) comprise indirect CO_2_ generated by the power plant supplying WRRF operation. They are defined by:

4 $${P}_{{CO}_{2},\;off-site}={k}_{PG}\cdot {e}_{D}$$

with the explained parameters in Listowski et al. (2011) and Mannina et al. (2016).

Off-site N_2_O emissions include N_2_O gas produced by biological degradation taking place in the effluent (Prendez & Lara-Gonzales, 2008; Mannina et al., 2016):

5 $${P}_{{N}_{2}O,\;off-site}= {N}_{effluent }\cdot {EF}_{effluent}$$

where *N*_*effluent*_ is the nitrogen load in the effluent and *EF*_*effluent*_ = 0.005·44/28 kg N_2_O/kg N (IPCC, 2006) is the emission factor for N_2_O emissions from the discharged wastewater.

The following expression is used to calculate the resource-removal section CO_2_ emissions:

6 $${P}_{{CO}_{2},\;on-site }={Q}_{influent}\cdot 0.99\cdot \left(1-{Y}_{H}\right)\cdot {\eta }_{ASP}\cdot bCOD+{Q}_{influent}\cdot 1.03\cdot {Y}_{H}\cdot {\eta }_{ASP}\cdot bCOD\cdot \frac{{k}_{d,H}\cdot MCRT}{1+{k}_{d,H}\cdot MCRT}$$

where explanations of the main used parameters can be consulted in Alex et al. (2008) and MCRT is the mean cell retention time, that is 15 days for this case study (Gori et al., 2011; Mannina et al., 2016).

The following relationship was used to estimate the on-site N_2_O emissions from the resource-removal section:

7 $${P}_{{N}_{2}O,\;on-site}={Q}_{influent}\cdot {(TN}_{in}-{TN}_{out})\cdot {r}_{{N}_{2}O}$$

where ${TN}_{in}$ represents the total nitrogen from the influent (kg N/m^3^), ${TN}_{out}$ is the total nitrogen in the effluent (kg N/m^3^) (Huang & Shen, 2019), ${r}_{{N}_{2}O}$ is the emission rate of N_2_O (kg N_2_O/kg N) (Baresel et al., 2016).

## Results and discussion

### Normal and faulty operation data sets

Six simulations were run to generate the data further used for PCA and FDA models development, one for fault-free functioning and five for faulty NO sensor. The plant model was simulated for 168 days. The quasi-steady state was achieved during the first 100 days of regular operation. Under the same operating conditions, data for normal operation was collected during the next 40 days. The malfunction cases were generated starting with the 140^th^ day of the simulations.

Data collected via simulation during the period of 40 days (i.e., days 100 to 139) of regular operation were recorded at every 15 min and used to generate the data matrix *X* of the PCA model of fault-free operation. Data generated during the period of 21 days (i.e., days 140 to 160) of NO sensor faulty operation were used to test the PCA model-based methodology for *fault detection* performance.

The data subsets used for training and testing the *fault discrimination* FDA model were also extracted from these sets. Data generated during the 141^st^ and 145^th^ days of fault-free and faulty NO sensor operation were utilized to train the model. The observation training matrix was formed of six classes (normal or fault-affected operation). The trained model was tested using data from the initial day of abnormal NO sensor operation, namely the 140^th^ day. This testing strategy was built to explore the FDA ability to diagnose the sensor malfunction within the first few hours following its starting action (i.e., the beginning of the day no. 140).

Twenty process variables were considered for the PCA model and twenty-one for the FDA model. They consist of secondary settler concentrations (3 variables), anoxic bioreactor (8 variables), aerated bioreactors (6 for PCA or 7 for FDA variables in total), mixed-liquor recycle flow rate (1 variable), aeration flow rate (1 variable) and one flow rate variable of the clean water effluent (1 variable). The variables considered were nitrate and nitrite nitrogen (*S*_*NO*_), free and saline ammonia (*S*_*NH*_), soluble biodegradable organic nitrogen (*S*_*ND*_), particulate biodegradable organic nitrogen (*X*_*ND*_), dissolved oxygen concentration (*S*_*O*_), readily biodegradable substrate (*S*_*S*_), slowly biodegradable substrate (*X*_*S*_), alkalinity (*S*_*alk*_), reactors flow rate (*Q*), mixed-liquid recycle flow rate (*Q*_*NR*_) and aeration flow rate (*Q*_*air*_). These variables were chosen based on the relative standard deviation values computed for the faulty time of operation.

### PCA model construction and fault detection results

The PCA model was built using 3840 observations, corresponding to the considered 40 days of regular operation for the 21 variables mentioned before. After scaling the training data matrix, matrices *T* and *P*, of scores and loadings were generated. A *CPV*_*k*_ threshold of 98.98% was selected. The number of principal components *k* was determined to be 8, taking into account the *CPV*_*k*_ values shown in Table S4 of SI. The scree test (Ledesma et al., 2015) confirmed the number of selected principal components, as can be observed in Fig. S3 of SI.

### Fault detection results

According to the PCA algorithm presented in Fig. S1 of SI, when a vector *x* of measured variables has a T^2^ or SPE value greater than the two corresponding thresholds, the presence of a NO sensor fault is detected.

The five different testing data matrices were formed using the vectors of faulty operation-measured variables. For every testing data matrix, a total of 2016 samples correspond to the simulated period from day 140 to day 160. For every sample vector of the testing matrices, the corresponding value of indices T^2^ and SPE was determined. They were compared with the threshold values for detecting the abnormal sensor activity. T^2^ and SPE values for the fault detection testing vectors were graphically presented together with ${T}_{\alpha }^{2}$ and *SPE*_*α*_ thresholds. They were able to reveal the presence of the NO sensor faults. The fault detection results of the investigated faults are presented in Figs. S4 to S9 of SI.

The detection of the NO sensor fault, based on the PCA model, proved to be efficient for the early detection of the NO sensor defects. The SPE fault detection methodology proved to have the best performance, when compared to the T^2^ one. However, they may be used simultaneously used to provide the consolidated assessment of fault occurrence and persistence.

**Fig. 2:**
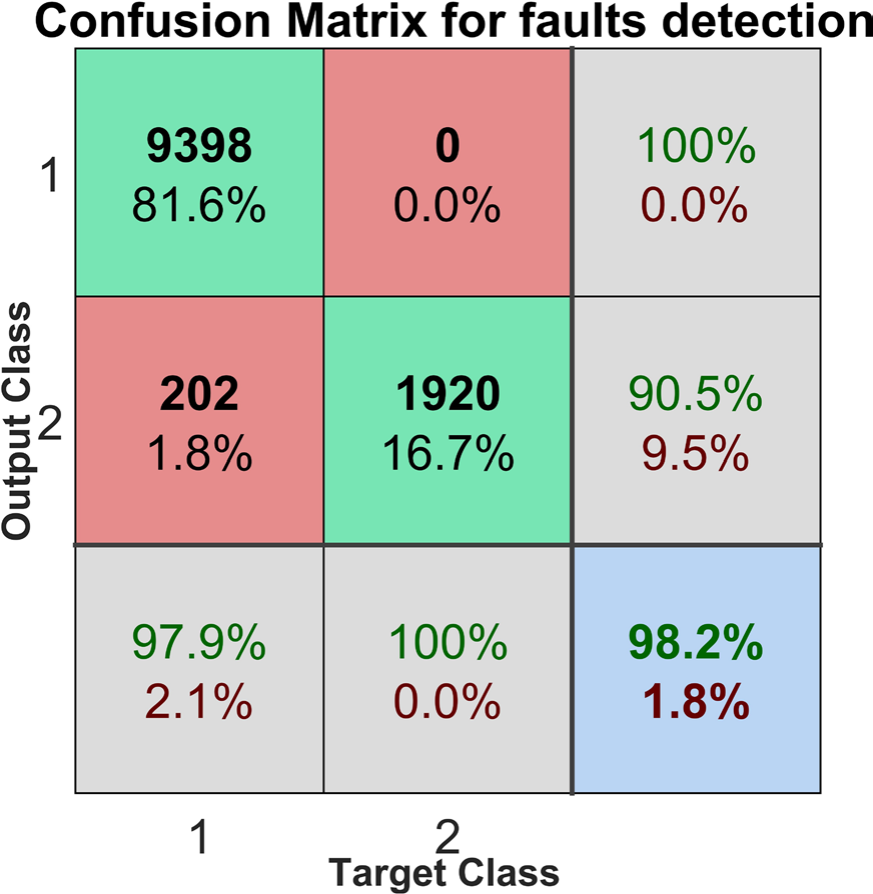
Confusion matrix of the fault detection

The emerged confusion matrix for the detection of the faults is presented in Fig. 2.

The confusion matrix for fault detection shows the evaluation of two classes: abnormal (with defect) and normal (no defect). The abnormal class appears in the first column (target), while the normal class is represented in the second column (target). The precision is 100% for the normal class prediction indicating no false positives and is 90.5% for the abnormal class. Only 202 observations were misclassified in the abnormal class, accounting for 1.8% of the total observations. The recall for abnormal class observations is 97.9%, meaning most true positives were detected, and recall for the normal class is 100%. FAR is null. Overall, the model achieved 98.2% accuracy, indicating strong performance in detecting correctly the observations.

The F1 score value for the abnormal class is 0.989, while F1 score for the normal class is 0.950. These scores reflect a strong balance between precision and recall for both classes, with the normal class performing particularly very well.

### FDA model construction and fault identification results

For each scenario, six distinct data classes were created: one for normal operation mode and five for the faulty NO sensor cases. These categories were built with data from days 141 to 145 and they served to train the FDA model. Each class was composed of a set of 480 observations. The observation training matrix for all classes had 2880 lines (observations) and 20 columns (variables). Data collected in the initial day of abnormal sensor operation, i.e. day 140, was used to test the trained FDA model's fault diagnosis performance. Each fault had 96 measurements in the testing data set. This testing strategy was devised to investigate the FDA model diagnosis ability to determine a specific kind of fault within a few hours of its appearance.

### Fault diagnosis results

The discriminant function values generated for every class were used to identify the sensor fault type. The highest value of the discriminant function revealed the corresponding fault class, and, diagnosed the fault type. The values of the discriminant functions *g*_*i*_ presented in SI were calculated for each of the 15-min time-sampled vector of measurements from the testing day, i.e. day 140, which were affected by the various types of faults (5 classes), as well as measurements corresponding to the normal operation (1 class). The values of the discriminant function that correspond to the first 24 h (testing day) of the normal and fault implementations are presented in Figs. 4, 5, 6, 7, 8 and 9. In these figures, all the discriminant functions *g*_*i*_ values of the investigated errors are represented using coloured curves. In each of these figures, the type of fault is identified by the curve (i.e. its associated fault type) that shows higher values than all other curves. This curve becomes dominant at a certain time point and remains dominant later on in the further time period. The point in time at which this curve becomes dominant over the others is considered the moment of the respective fault identification. On each figure, a subplot was added to present a detailed zoom for the ordinate axis, better revealing and visualizing the time period in the plot where the dominance of the type identifying *g*_*i*_ function occurred.

The confusion matrix was also built for the FDA identification method. In this matrix the normal class (fault-free operation, target 1) is presented in the first column. It is followed by the faulty classes (targets) in the next columns, with the order: constant additive error (target 2), Ramp changing error in time (target 3), incorrect amplification (target 4), random additive error (target 5) and unchanging sensor value (target 6).

**Fig. 3:**
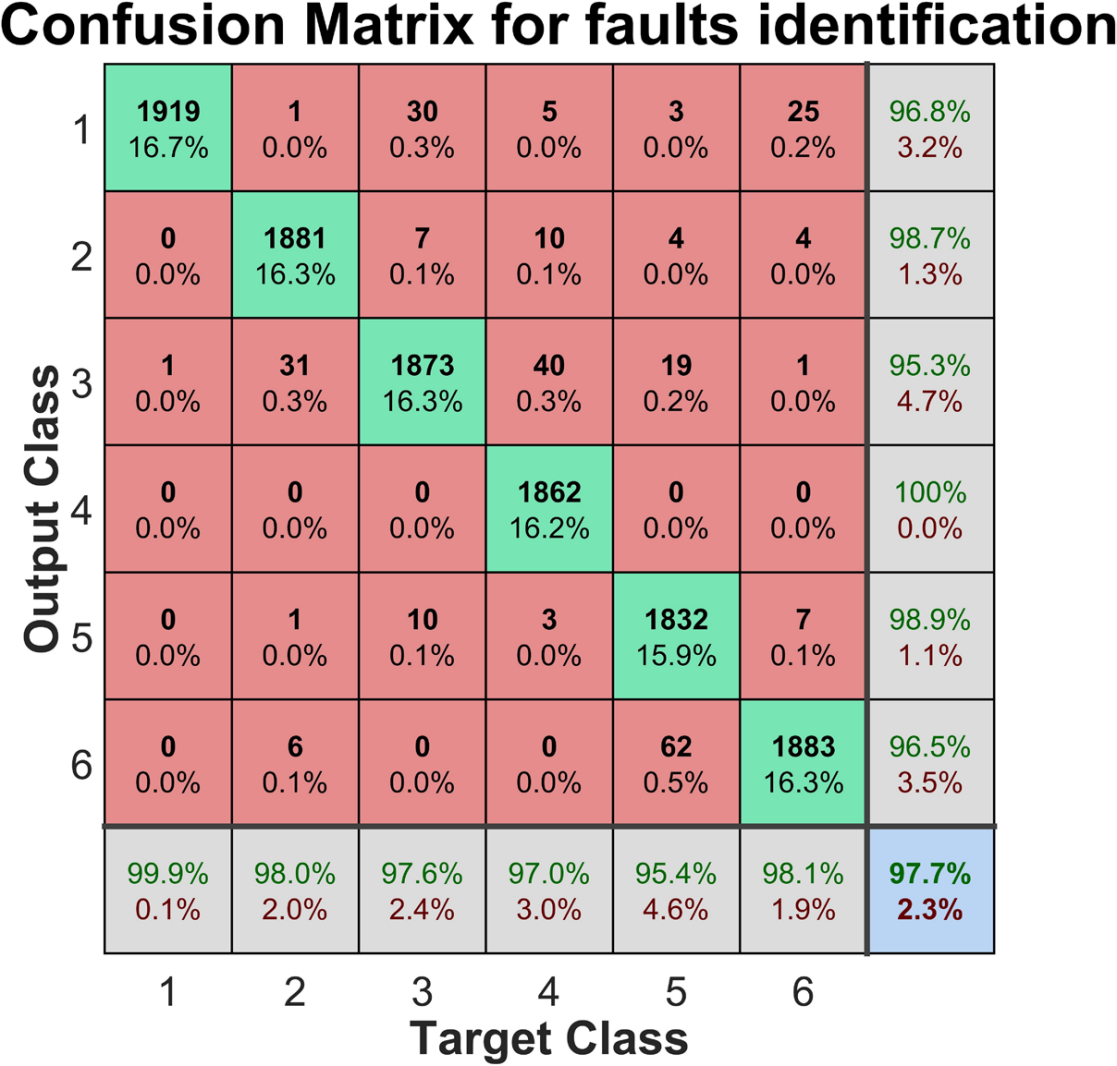
Confusion matrix of the fault identification

As it can be observed in Fig. 3, the overall accuracy is of 97.7%.

**Fig. 4:**
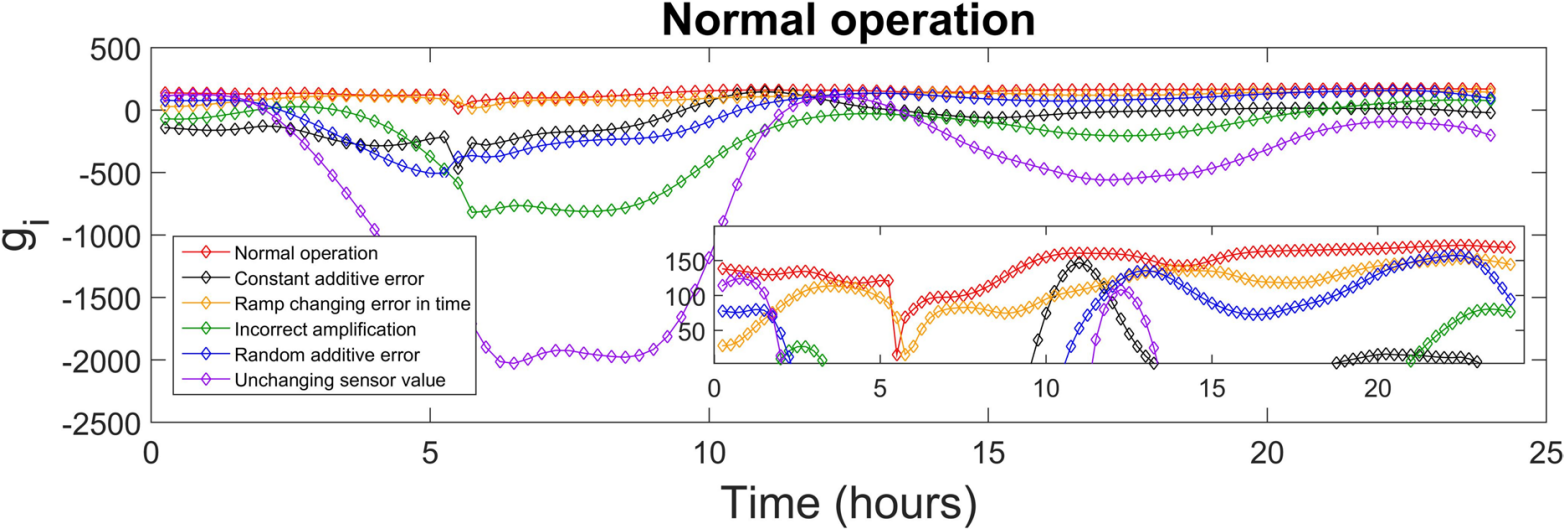
Normal operation diagnosis

As shown in Fig. 4, the *g*_*i*_ values that correspond to the normal operation curve, represented with red colour on the graph, identified the fault-free normal operation. From the beginning of the 24 h of observation and except for one single value, at time 5.5 h, all points of the red curve had the largest values during the testing day, recognizing the normal operation. In this scenario, the long-term precision was 96.8%, with a recall value of 99.9%. The F1 score was calculated to be 0.983, providing a balanced measure of precision and recall. The MAR value is low in this case, with the value of 0.1%.

**Fig. 5:**
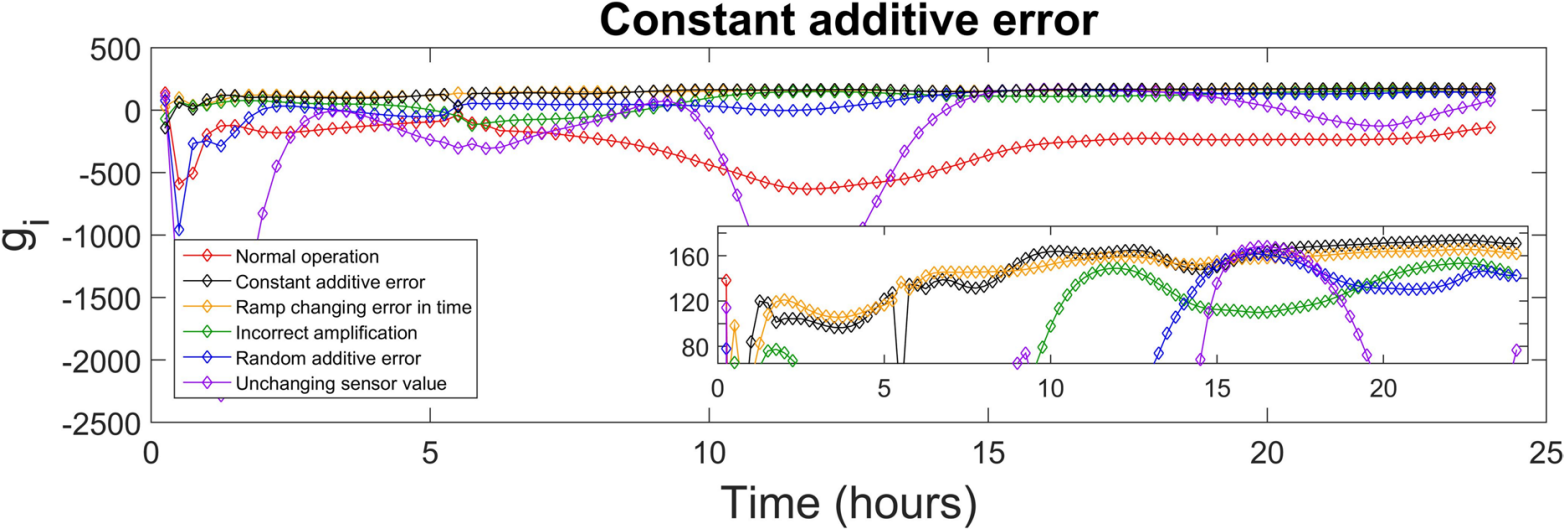
Constant additive error diagnosis

Figure 5 shows that constant additive error is effectively diagnosed for the time periods: 1–1.5 h, 5–5.25 h, 5.75 h, 8.75–13.75 h. Then, from 17 h until the end of the day and for next period of time, the black curve becomes dominant, indicating the correct identification of the constant additive class of error with a long-term precision of 98.7%.

At earlier time points and between the specified intervals, the constant additive error FDA model indicated normal operation or misclassified the error as a random additive error in 25 measurements, resulting in a MAR of 2%. The F1 score for this classification scenario was 0.983, reflecting a good detection of the error type.

**Fig. 6:**
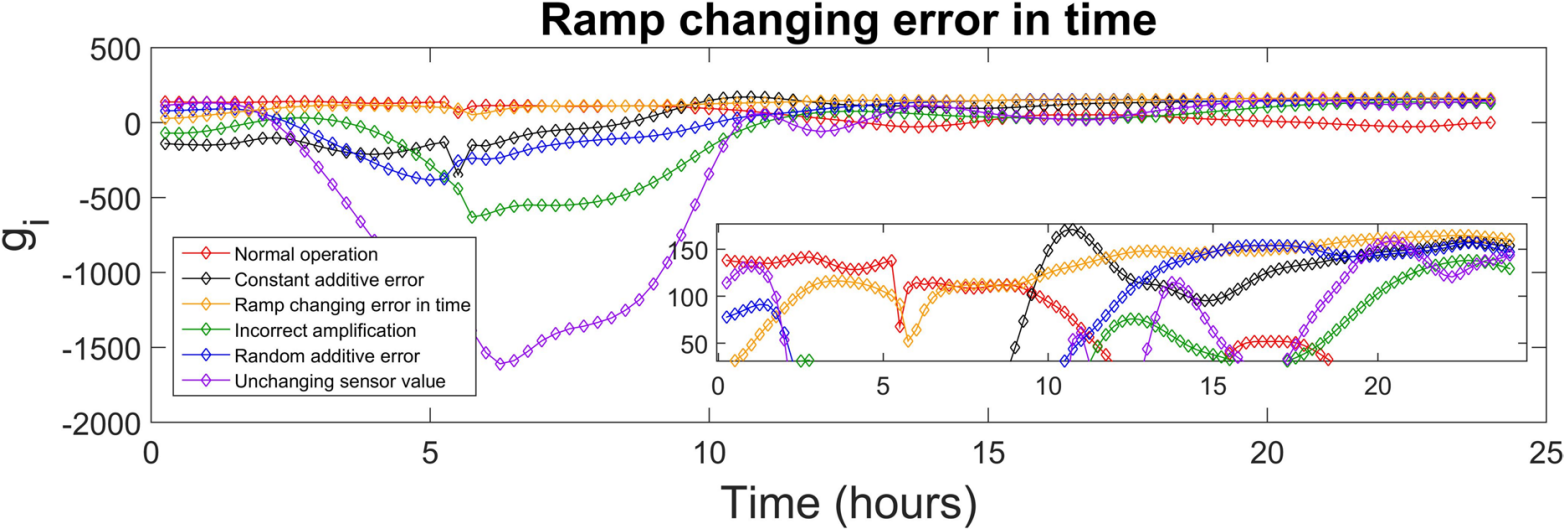
Ramp changing error in time diagnosis

Ramp changing error in time diagnosis is confirmed at time point 5.5 h, then between 7.25–8.25 h, 9–9.5 h, 11.75–14.75 h and finally from 18 h until end of the day. As shown in Fig. 6, the yellow curve becomes dominant in comparison with the other curves after 18 h. Ramp changing error in time has the magnitude increasing over time and its effects follow this trend due to the intrinsic nature of this error. During the initial moments following the fault’s appearance and for brief intervals, this error is interpreted as either constant additive error or random additive error. In this case, the long-term precision is 95.3%, while the recall stands at 97.6%. The F1 score is 0.964 and MAR has a value of 2.4%, indicating a good detection performance for the ramp changing error in time.

**Fig. 7:**
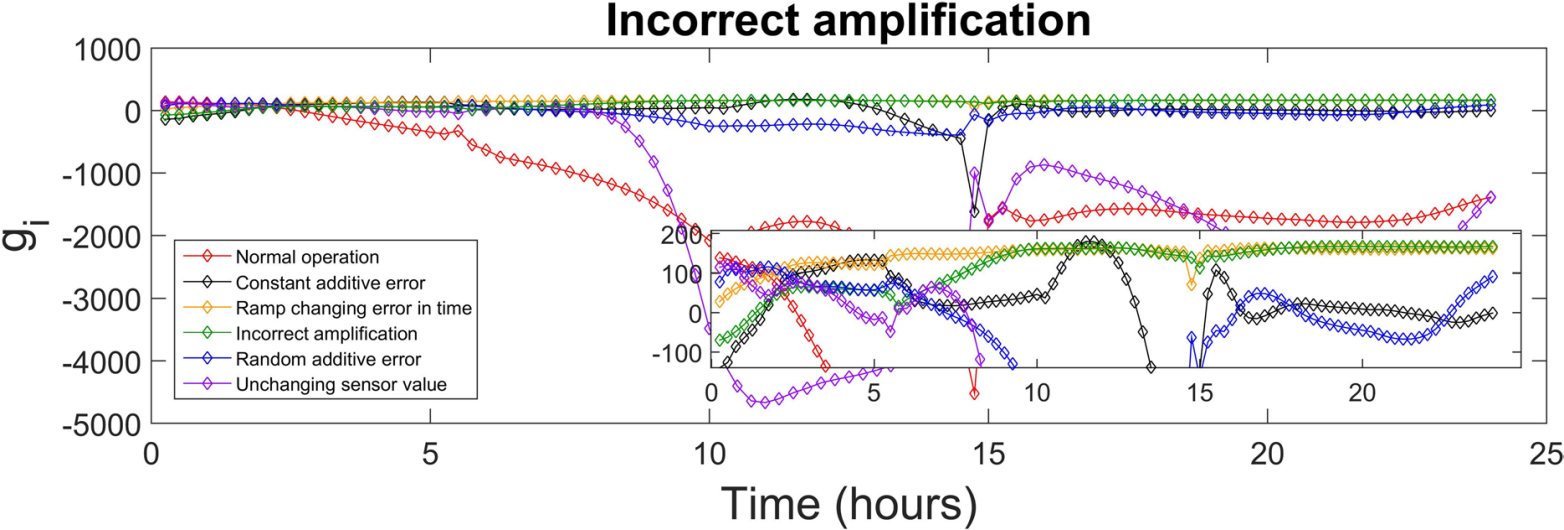
Incorrect amplification diagnosis

The incorrect amplification fault diagnosis is presented in Fig. 7. This type of fault is properly and firmly diagnosed after 17.75 h, but it is also correctly diagnosed three times before that time moment, i.e. in the 9.75–11-h and 12.25–12.75-h time intervals and at the time moment of 14.75 h. The green curve that represents the incorrect amplification discriminant values becomes and remains dominant after the time moment of 17.75 h. This results in a long-term precision of 100%, with a value of the recall of 97.0% and a MAR of 3%. The F1 score is 0.985, reflecting a very good capacity for identifying the fault.

**Fig. 8:**
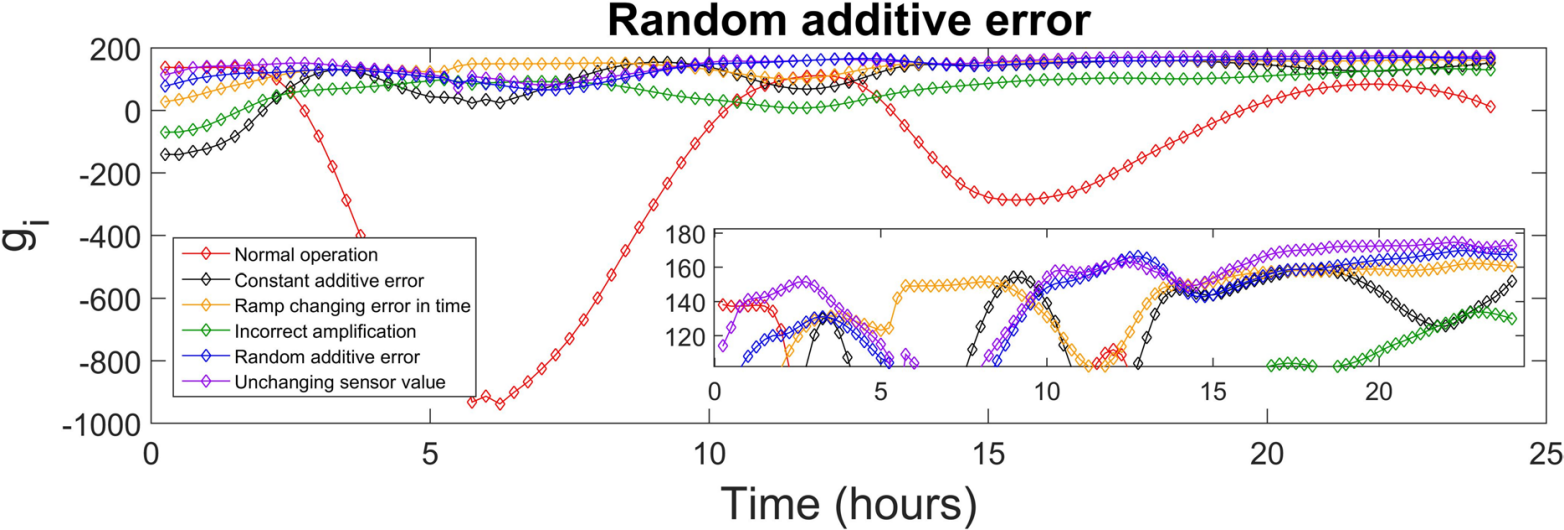
Random additive error diagnosis

Random additive error is correctly diagnosed between 12 and 13.75 h, as presented in Fig. 8. During the first day, the fault was temporarily misdiagnosed either as an unchanging sensor value error or as a ramp-changing error. The long-term precision for this fault is 98.9% and the recall value is 95.4%, indicating a good proportion of correct identifications. The F1 score is 0.971 and MAR is 1.1%, reflecting a good ability to accurately detect the random additive error.

**Fig. 9:**
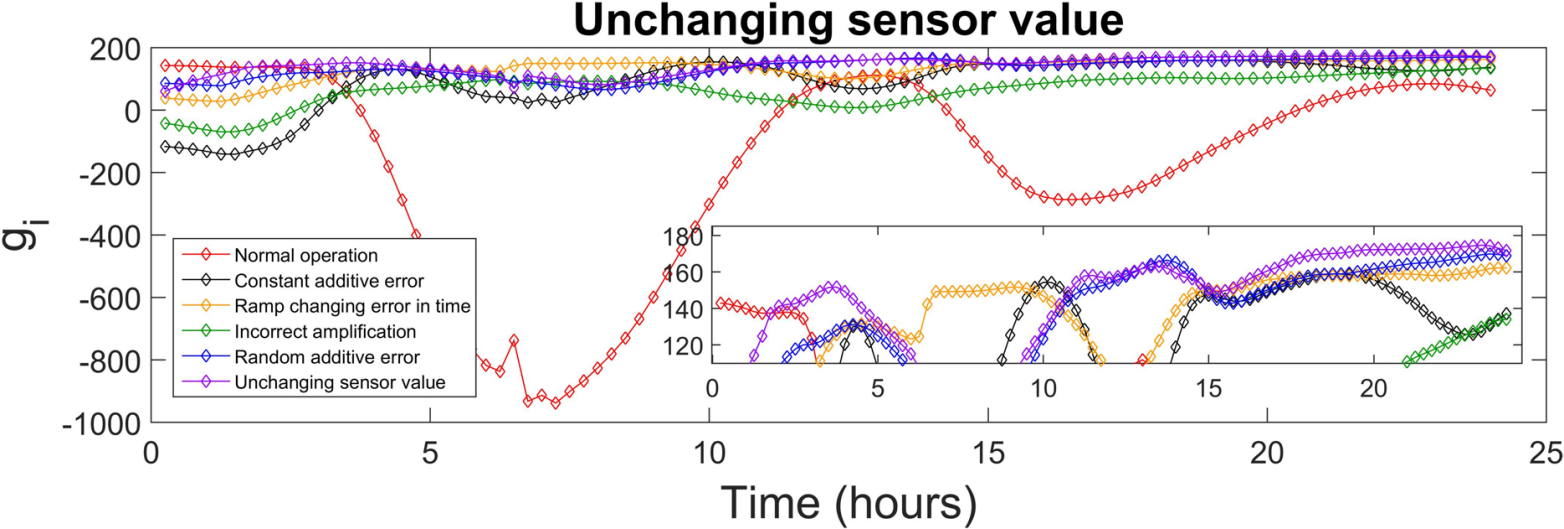
Unchanging sensor value diagnosis

The unchanging sensor value fault type is diagnosed and evaluated, and results are presented in Fig. 9. It is correctly diagnosed between 2–5.25 h, 10.75–12.75 h, 14.75–15 h and then, is persistently and rightly diagnosed after the time moment of 15.5 h. As shown in Fig. 9, the purple curve representing this fault type becomes dominant after 15.5 h, indicating successful identification. For the instances where this fault was misdiagnosed, the MAR is 1.9%. The F1 score of 0.973 and the long-term precision of 96.5% reflect a strong ability to correctly identify the unchanging sensor value fault.

Some of the discriminant function values are similar due to the extremely close values of the process measured variables, caused by comparable values of the NO sensor signals emerged from the considered fault scenarios. This contributes to misdiagnosis of some faults for the very close moments or period after the fault occurrence and for longer or shorter time periods afterwards.

Despite these misclassifications, the faults are persistently and firmly identified during the very first day (one group before 5–6 h and one group before 11–12 h). The evaluation of fault detection and identification methods showed good performance across all fault types and normal operation. This is a valuable result, as the failures identification is very difficult to be unveiled by the operator but can be systematically and efficiently assessed in a period of several hours, before their induced effects aggravate the performance operation. Coupled with the PCA procedure, the FDA methodology may become an opportune support tool for maintaining efficiency in the management of the WRRF.

### Performance evaluation of the fault-affected WRRF

Performance indices AE, PE and EQ were computed and averaged over the 28 days of normal and faulty operation cases. Table 1 presents the AE, PE and EQ mean values for regular and sensor failure operation.

**Table 1:** Assessment of the performance indices for the regular and faulty process cases

Operating case	AE (kWh/day)	PE (kWh/day)	Total energy demand (kWh/day)	EQ (kg PU/day)
Normal operation	16,992	1329	18,321	16,852
Constant additive error	17,253	1633	18,886	15,050
Ramp changing error in time	17,776	2320	20,096	14,911
Incorrect amplification error	17,496	1956	19,452	17,108
Random additive error	17,752	2232	19,984	17,186
Unchanging sensor value error	17,848	2415	20,263	17,198

In terms of AE, PE and total energy demand, all faults have a negative impact and contribution to the higher energy consumption. A faulty value of the NO sensor signal determines an incorrect mixed-liquor recycle flow that needs to be pumped. This circumstance is followed by disturbed concentrations of NO and DO in the aerobic reactor. As a result, the DO controller drives the aeration in the aerated reactors to inefficient values of the air flow rate, with direct consequences on the increase of the AE. Incorrect amplification, random additive, and unchanging sensor value errors induce the deterioration of the EQ. Constant additive error and ramp changing error in time have positive impacts on the EQ index, but with the expense of higher total energy demand and potential undesired long-term effects. The dominant increase in the total energy demand is aggravated by the negative influence of the GHGs emitted by the combustion of conventional fuels requested for the production of the WRRF externally originating electrical energy.

When positive values for the c constant were considered to the constant additive and to the ramp changing error types of faults, both the aeration energy and pumping energy decreased due to the intervention of the NO concentration control loop, but the quality of the clean effluent deteriorated. It is, for example, the case of the biomass that shows the decrease of both heterotrophic and autotrophic species concentration. They furthermore induced the depreciation of the clean effluent quality. A similar negative impact was noted on GHG emissions.

### Energy costs assessment for faulty operation cases

The WRRF necessitates a substantial energy supply, as well as significant economic costs. Geopolitics, the regional specific power mix, supply chain expenses, environmental protection taxes, extreme weather, excise or taxation rates have a direct impact on energy prices. The type of energy source also influences the cost of energy. A comparison of the energy prices, depending on the source or technology used to produce it, is presented in Table S5 of SI.

According to Table S5, prices of energy range from 4.8 to 12.10 eurocents. These figures originate from an International Energy Agency (IEA) report on average energy costs. This report included data on 243 plants from 24 European, African and Asian countries (IEA, 2023).

The daily cost for normal and for each type of fault affected operation was determined. The daily costs of various potential electricity sources or energy-generating technologies were specifically assessed. The findings are summarized in Table S6 of SI.

Analysing the expenses of energy spent in various fault cases it is clear that unchanging sensor value fault implies the highest energy costs and the constant additive error implies the smallest energy cost.

As expected, the daily energy costs values shown in Table S6 disclose that onshore wind, solar PV commercial, and hydro run of rivers are the most cost-effective sources of power to use, while solar PV residential, solar thermal (CSP) and biomass are the costliest. The costs of lignite (CSS) and coal (CSS) energy sources may also be considered as high costs.

Switching to lower priced energy sources and integrating a system tailored to use a combination of energy sources in cases of the occurrence of a sensor failure may lower the implied electrical energy expenses.

### Environmental assessment of CO_2_and N_2_O emissions

The on-site and off-site CO_2_ and N_2_O emissions were calculated for both, normal and faulty NO sensor operation. Table 2 shows the daily mean values obtained for the investigated cases.

**Table 2:** Emissions due to the NO sensor defects

Emissions type	Process	Emitted gas	Type of fault					
			Normal operation	Constant additive error	Ramp changing error in time	Incorrect amplification error	Random additive error	Unchanging sensor value error
Off-site emissions	Power generation	CO_2_, ${P}_{{CO}_{2},off-site}$, kg CO_2_/day	3481	3588	3818	3696	3797	3850
	Biological degradation in the WRRF effluent	N_2_O, ${P}_{{N}_{2}O,off-site}$, kg N_2_O/day	3.61	3.65	3.50	3.63	3.51	3.47
On-site emissions	Resource-removal section biological processes	CO_2_, ${P}_{{CO}_{2},on-site}$, kg CO_2_/day	13,689	13,869	14,175	14,037	14,137	14,207
		N_2_O, ${P}_{{N}_{2}O, on-site}$, kg N_2_O/day	10.35	10.34	10.40	10.35	10.40	10.42
Total emissions		CO_2_, ${P}_{{CO}_{2}, total}$, kg CO_2_/day	17,170	17,457	17,993	17,733	17,934	18,057
		N_2_O, ${P}_{{N}_{2}O, total}$, kg N_2_O/day	13.96	13.99	13.90	13.98	13.91	13.89
		CO_2e_, ${P}_{{CO}_{2e},overall}$, kg CO_2e_/day	21,330	21,626	22,135	21,899	22,079	22,196

The data presented in Table 2 shows that on-site emissions, ${P}_{{CO}_{2},on-site}$ and ${P}_{{N}_{2}O,on-site}$, are the most significant, accounting for about 75–80% of the total emission. ${P}_{{CO}_{2},total}$ prevails over ${P}_{{N}_{2}O,total}$ values, for faulty and normal operation. Computed overall equivalent CO_2_ emissions ${P}_{{CO}_{2},overall}$ showed increased values for all cases of NO sensor faults. The unchanging sensor value type of fault resulted in the highest overall equivalent CO_2_ emissions. The constant additive error had the lowest emission values. Incorrect amplification had the next higher overall equivalent CO_2_ emissions.

The N_2_O emissions showed reduced variations. The assessment of total N_2_O emissions for the constant additive error and incorrect amplification fault showed marginally increased values for total N_2_O emissions. For other faults, these values are slightly lower than the normal case values due to the very small reduction of the effluent nitrogen load.

The assessment of CO_2_ and N_2_O emissions caused by various defects of the NO sensor provides useful quantitative assessment data regarding the most disadvantageous faults. This evaluation serves as the groundwork for the design of control and safety solutions aimed at ensuring the process sustainability goal.

## Conclusions

In this paper, the PCA-based sensor fault detection methodology along with FDA-based sensor fault identification approach were investigated for the NO sensor of the urban WRRF. Constant additive error, ramp changing error in time, incorrect amplification error, random additive error and unchanging sensor value error were the examined faults. The data sets associated with regular and defective operation were generated using a calibrated WRRF model. This study was performed in the configuration that used two main control loops. The first control system aims to control NO concentration in the anoxic reactor, while the second one controls the concentration of DO in the aeration bioreactors. The considered NO sensor is an essential component of the control loop responsible for the WRRF denitrification step. Its performance is both directly and indirectly determining the total amount of energy spent by the WRRF and influences the economic costs. Furthermore, the efficiency of the denitrification process has a significant impact on the quality of effluent water and GHG emissions. The conclusions are presented on three categories of results.

First, the PCA detection algorithm performance was investigated, and main drawn conclusions are:

- The detection of faults was very good, as the detection accuracy reached the value of 98.2%, and the detection was prompt, as it was typically achieved in less than 3.75 h;
- The constant additive error and unchanging sensor value type of faults were detected in 1.5 h, demonstrating the fastest detection capability;
- The ramp changing error was identified the latest, i.e. after 19.25 h, performance attributed to its gradually increasing nature which hinders rapid detection;
- The SPE method proved to be more efficient and accurate than Hotelling’s T^2^ detection approach.

Second, the FDA fault diagnosis methodology was used for the identification of five types of faults, and the obtained results derived the following main findings:

- The overall diagnosis accuracy was of 97.7% and the diagnosing precision was higher than 95.3% for all investigated types of fault;
- The short-time diagnosis of fault types ranged from 1 to 12 h, and the long-time consolidated identification varied between 5.5 and 18 h, revealing differences among the fault types;
- The constant additive error was the most rapidly diagnosed, i.e. within 1 h, being followed by the unchanging senor value, incorrect amplification, ramp changing and random additive types of error.

Third, the study proposed and assessed the performance of the WRRF, i.e. effluent quality EQ, aeration AE and pumping PE spent energy, and greenhouse gas GHG emissions, for the operation of the plant in the presence of the different NO sensor types of fault. This investigation led to the following conclusions:

- When compared to the normal operation, the EQ index value depreciated to varying degrees during operation with incorrect amplification and random additive error, revealing that unchanging sensor value error was the most detrimental fault to the EQ index, and that constant additive error and ramp changing error in time determined a reduction of the EQ value;
- The sum of the AE and PE index showed an increased spent energy value of up to 10.5% during operation with all sensor types of fault, revealing the highest percentual increase for the PE, a result which may be attributed to the direct effect of the NO sensor faults on the WRRF internal recycle flowrate;
- The environmental impact of the NO sensor faults on both on-site and off-site CO2 and N2O emissions, disclosed that the primary source of GHGs is the on-site emissions, accounting for roughly 75% of total GHG emissions. While all faulty cases showed a negative effect on the GHG emissions, the case with the greatest unfavourable effect, of about 4 % increase, was the unchanging sensor value type of fault.

The results obtained by the investigations performed on the NO sensor faults detection and diagnosis also revealed some efficiency limitations of the proposed methodologies to the cases when insufficient, inappropriate or imprecise data sets are used to build the PCA-based models for faults detection and identification. Future direction of study is envisioned for investigating the detection and diagnosis methodologies and performance of the WRRF operation in the presence of the simultaneous faults affecting the Dissolved Oxygen and NO sensors.

The current research results are significant on different levels of the potential users, beyond the WRRF application. At the instrumentation level, the implemented detection and diagnosis presented algorithms may be used in the instrumentation maintenance tasks for promptly indicating sensors affected by incipient or fully developed malfunctioning state. The proposed methodology can be extended to other instrumentation equipment, such as control valves, controllers and software components of the soft controllers. At the same level, the sensor manufacturers can extend the software modules of their products with self-diagnosing components based on the faults’ detection and identification methods. Coupled with adaptive learning capabilities, the intelligent sensors may be further extended. At the application level, the safe and efficient operation of the plant may benefit from the early detection of the sensor faults, highlighting the quantitative impact of the sensor fault type on the spent energy and product quality, consequently improving plant management. At the sustainable development level, the quantitative evaluation of the defective sensor’s influence on the environment will offer important information both for taking decisions to limit or prevent accidental pollution of water, air and soil, and for the comprehensive integrated plant and control system design.

## Supplementary Information

Below is the link to the electronic supplementary material.

ESM 1 (DOCX 1.81 MB)

## Data Availability

No datasets were generated or analysed during the current study.
