# Supplementary material for: Fault detection and diagnosis of the wastewater nitrate and nitrite sensors using PCA and FDA combined with assessment of the economic and environmental impact of the faults
Source: Environ Monit Assess. 2025 Jan 2;197(1):121. doi: 10.1007/s10661-024-13593-z (PMC11695568; doi:10.1007/s10661-024-13593-z)
Supplement: Supplementary file 1 — (DOCX 1.81 MB) [file 10661_2024_13593_MOESM1_ESM.docx]

**Supplementary Information (SI)**

**Fault detection and diagnosis of the wastewater nitrate and nitrite sensors using PCA and FDA combined with assessment of the economic and environmental impact of the faults**

Alexandra-Veronica Luca^1^, Melinda Simon-Várhelyi^1^, Norbert-Botond Mihály^1^, Vasile-Mircea Cristea^1*^

[alexandra.luca@ubbcluj.ro](mailto:alexandra.luca@ubbcluj.ro), [melinda.varhelyi@ubbcluj.ro](mailto:melinda.varhelyi@ubbcluj.ro), [norbert.mihaly@ubbcluj.ro](mailto:norbert.mihaly@ubbcluj.ro)

^1^ Department of Chemical Engineering, Faculty of Chemistry and Chemical Engineering,

Babes-Bolyai University of Cluj-Napoca, 11 Arany János Street, 400028 Cluj-Napoca, Romania

*Corresponding author: [mircea.cristea@ubbcluj.ro](mailto:mircea.cristea@ubbcluj.ro); Tel.: +40 264 593833

Table S1 contains information about the design characteristics and dimensions of WRRF equipment.

Table S1. Main equipment parameters of the municipal WRRF

| Variable | Value | Measurement unit |
| --- | --- | --- |
| Primary settler | | |
| Area | 2,125 | m^2^ |
| Height | 3.5 | m |
| Anaerobic bioreactor | | |
| Volume | 9,015 | m^3^ |
| Anoxic bioreactor | | |
| Volume | 12,678 | m^3^ |
| Aerobic bioreactors | | |
| Volume | 33,066 | m^3^ |
| Area | 6,012 | m^2^ |
| Secondary settler | | |
| Area | 11,304 | m^2^ |
| Height | 3 | m |

The average values of the influent, effluent and main operating variables are presented in Table S2.

Table S2. Average values of the main influent, operating and effluent variables of the municipal WRRF

| Variable | Average value | Measurement unit |
| --- | --- | --- |
| Influent data | | |
| Influent flow rate | 116,300 | m^3^/day |
| Chemical oxygen demand | 279.11 | g COD/m^3^ |
| Free and saline ammonia | 26.24 | g N/m^3^ |
| Organic nitrogen | 7.91 | g N/m^3^ |
| Alkalinity | 7.68 | - |
| Total suspended solids | 137.63 | g SS/m^3^ |
| Temperature | 16.65 | ^o^C |
| Operating data | | |
| Total air flow rate | 297,293 | m^3^/day |
| Nitrate recycling flow rate | 107,380 | m^3^/day |
| Return activated sludge flow rate | 112,500 | m^3^/day |
| Waste flow rate | 890 | m^3^/day |
| Effluent data | | |
| Effluent flow rate | 115,410 | m^3^/day |
| Chemical oxygen demand | 21.50 | g COD/m^3^ |
| Free and saline ammonia | 0.19 | g N/m^3^ |
| Nitrate and nitrite | 2.21 | g N/m^3^ |
| Organic nitrogen | 1.83 | g N/m^3^ |
| Alkalinity | 5.61 | - |
| Total suspended solids | 12.10 | g SS/m^3^ |

Sensors faults and their implementation

The mathematical form of the considered faults is presented in Table S3, where *u(t)* denotes the NO concentration variable, *y(t)* represents the output signal of the sensor, *c* designates either a constant or a random time varying value involved in the faulty signal generation and *t* is time.

Table S3. Mathematical expressions of the implemented fault types

| Fault type | Fault expression |
| --- | --- |
| Fault-free operation (normal) | y(*t*) = u(*t*) |
| Constant additive error (bias) | y(*t*) = u(*t*) + *c*, *c* = constant |
| Ramp changing error in time (drift) | y(*t*) = u(*t*) + (*t*-*t_0_*)·*c*, *c* = constant |
| Incorrect amplification (wrong gain) | y(*t*) = u(*t*)·*c*, *c* = constant |
| Random additive error (loss of accuracy) | y(*t*) = u(*t*)+c(*t*), *c*(*t*) = uniform random value |
| Unchanging sensor value (fixed value) | y(*t*) = *c*, *c* = constant |

Principal Component Analysis algorithm

Consider *X*, the original data matrix of *m* lines and *n* columns, with *m* representing the samples number and *n* denoting the process variables’ number corresponding to a regular, fault free process:

| *X =* $\left[ \begin{matrix} \begin{matrix} x_{11} & x_{12} \\ x_{21} & x_{22} \end{matrix} & \cdots& \begin{matrix} x_{1n} \\ x_{2n} \end{matrix} \\ \vdots& \ddots& \vdots\\ x_{m1} x_{m2} & \cdots& x_{mn} \end{matrix} \right]$ | (S1) |
| --- | --- |

After the normalisation of the original data matrix *X* to zero mean and unit variance, the matrix $\dot{X}$is obtained. Subsequently, matrix *C* can be computed:

| *C* *=* $\frac{1}{m - 1}$ *·*$\dot{X}^{T}$*·*$\dot{X}$ | (S2) |
| --- | --- |

and

| *C = V · S · V^T^* | (S3) |
| --- | --- |

where *V* is the matrix of which columns are the eigenvectors of the covariance matrix *C* and *S* is the diagonal matrix that contains the square roots of the eigenvalues of *C* arranged in decreasing order of their magnitude. The loadings matrix named *P* is formed from the first *k* columns of *V* that correspond to the first *k* principal eigenvalues. The following validation can be made for the reconstruction of the normalized data matrix:

| $\dot{X}$ *=* ${T\cdot P}^{T}$*+ E =* ${t_{1}{\cdot p}_{1}}^{T}$ *+* ${t_{2}{\cdot p}_{2}}^{T}$ *+ … +* ${t_{k}{\cdot p}_{k}}^{T}$ *+ E* | (S4) |
| --- | --- |

where *T* is the score matrix and *E* represents the residual matrix.

The value of cumulative percent variance (CPV_k_) reflects the percentage of variance captured by the first *k* principal components. Its mathematical expression is:

| *CPV_k_ =* $\frac{\sum_{j=1}^{k} \lambda_{j}}{\sum_{j=1}^{m} \lambda_{j}}$ *· 100* | (S5) |
| --- | --- |

where $\lambda_{j}$ is the *j*^th^ eigenvalue of the covariance matrix, *C*.

Hotelling’s T^2^

The multivariate Hotelling’s T^2^ offers a complete measure of the variations in PCS. For a sample vector x, its mathematical expression is:

| *T^2^ =* *x^T^ · P ·*$S_{k}^{-1}$*· P^T^ · x* | (S6) |
| --- | --- |

where *S_k_* is formed by the first *k* lines and columns of the matrix *S*.

The value of the threshold $T_{\alpha}^{2}$ is the indicator of the process state. If the value *T^2^* is higher than the threshold $T_{\alpha}^{2}$, the process is faulty, otherwise it is normal. $T_{\alpha}^{2}$ depends on the critical value of the F-distribution, namely on $F_{\alpha}$*(k, m* $-$*k)*. *α* represents the level of confidence with values ranging from 90% to 95% and *k*, *m*$-$*k* are the degrees of freedom. $T_{\alpha}^{2}$ can be calculated as it follows:

| $T_{\alpha}^{2}$*=* $\frac{{(m}^{2} - 1) \cdot k}{m \cdot(m - k)}$*·*$F_{\alpha}$*(k, m*$-$*k)* | (S7) |
| --- | --- |

Square Prediction Error

SPE measures the variations in the RS as a sum of the residuals squares:

| $SPE$*=* $r^{T}\cdot r$ | (S8) |
| --- | --- |

with *r* the residual vector:

| $r=x-\dot{x}$ *= (I*$-$*P · P^T^) · x* | (S9) |
| --- | --- |

where *I* is the identity matrix. The threshold ${SPE}_{\alpha}$ depends on the standard normal deviation value for *1*$-$*α* percentile, $C_{\alpha}$:

| ${SPE}_{\alpha}$ *= θ_1_*$\cdot\left[ \frac{h_{0}\cdot C_{\alpha} \cdot\sqrt{2 \cdot\theta_{2}}}{\theta_{1}}+1+\frac{\theta_{2}\cdot h_{0} \cdot(h_{0} - 1)}{\theta_{1}^{2}} \right]^{\frac{1}{h_{0}}}$ | (S10) |
| --- | --- |

where

| $h_{0}=1-\frac{2{\cdot\theta}_{1}{\cdot\theta}_{3}}{3{\cdot\theta}_{2}^{2}}$ | (S11) |
| --- | --- |

and

| $\theta_{i}$ *=* $\sum_{k+1}^{n} \lambda_{k+1}^{i} for i=1, 2, 3$ | (S12) |
| --- | --- |

As in the $T_{\alpha}^{2}$ case, a higher value of $SPE$ than the threshold ${SPE}_{\alpha}$ indicates an abnormal operation.

The flow diagram of the PCA fault detection algorithm is given in Fig. S1.


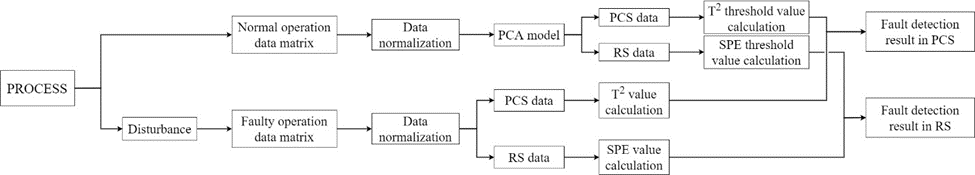


**Fig. S1** Flow diagram of the PCA algorithm

Fisher Discriminant Analysis method

For the previously defined matrix ${X\in R}^{m\times n}$ which encapsules the training information applicable to all classes of both normal and faulty operation, the submatrix $X_{i}$ is the measurements’ subset that consists of $n_{i}$ rows and corresponds to the category of samples named $i$. $\overline{x_{i}} i$s denoting the *m*-dimensional sample mean vector for the class $i$:

| $\overline{x_{i}}=\frac{1}{m_{i}}\sum_{x_{j}\in X_{i}} x_{j}$ | (S13) |
| --- | --- |

where $x_{j}$ is the group of vectors which correspond to class *j*. To define the within-class-scatter matrix the following relation is used:

| $S_{w}=\sum_{i=1}^{c} S_{i}$ | (S14) |
| --- | --- |

with *c* the number of classes. The within-scatter matrix for class $i$ is defined by:

| $S_{i}=\sum_{x_{j}\in X_{i}} (x_{j} -\overline{x_{i}}){(x_{j} -\overline{x_{i}})}^{T}$ | (S15) |
| --- | --- |

The between-class-scatter matrix is then given by:

| $S_{b}=\sum_{i=1}^{c} m_{i} (\overline{x_{i}}-\overline{x})(\overline{x_{i}}-\overline{x})^{T}$ | (S16) |
| --- | --- |

with $\overline{x}$ the overall mean vector of all average values of the columns of $X$.

The Fisher criterion is maximized to determine the optimal discriminant direction.:

| $J\left( \varphi\right)=\frac{\varphi^{T}S_{b}\varphi}{\varphi^{T}S_{w}\varphi}$ | (S17) |
| --- | --- |

where the maximiser $\varphi$ represents the optimal discriminant direction that maximizes the ratio of the between-class scatter to the within-class scatter. The vector $\varphi$ that maximizes $J\left( \cdot\right)$needs to satisfy the formula:

| $S_{b}\varphi=\lambda S_{w}\varphi$ | (S18) |
| --- | --- |

for a constant $\lambda$ that denotes the distinction between categories. If $S_{w}$ is nonsingular, it is attained as the following expression of conventional eigenvalue problem:

| $S_{w}^{-1}S_{b}\varphi=\lambda\varphi$ | (S19) |
| --- | --- |

The sum of $S_{b}$ and $S_{w}$ gives the total-scatter matrix:

| $S_{t}$*=*$S_{b}$*+*$S_{w}$ | (S20) |
| --- | --- |

If vector $x_{j}$ implied by the *m*-dimensional space is converted to the *a*-dimensional space determined by the Fisher Discriminant Analysis vectors, then its linear transformation can be obtained using:

| $z_{i}$*=*$W_{a}^{T}x_{j}$ | (S21) |
| --- | --- |

where $W_{a}^{T}$has the *a* FDA vectors as columns and $z_{i}$ ${\in R}^{a}$*.*

To identify the errors, the Fisher Discriminant Analysis investigates the observations gathered under multiple faulty conditions and employs a discriminant algorithm to identify similarities between the existing data and the data associated to each class. The observation is assigned to class *I* when the maximum discriminant function value, $g_{i}$, meets the following conditions:

| $g_{i}$*(x) >* $g_{j}$*(x), Ɐ j ≠ i* | (S22) |
| --- | --- |

The observed vector *x* of class *i* gives the discriminant function, $g_{i}$*(x)*, and the observed vector *x* of class *j* provides the discriminant function,$g_{j}$*(x)*. The discriminant function may be easily determined, for each group *i*, with the formula listed below:

| $g_{i}$*(x) =*$-$ $\frac{1}{2}(x-\overline{x_{i}})^{T}W_{a}\left( \frac{1}{m_{i}-1}W_{a}^{T}S_{i}W_{a} \right)^{-1}W_{a}^{T}\left( x-\overline{x_{i}} \right)+ln--\frac{1}{2} ln\left[ det( \frac{1}{m_{i}-1}W_{a}^{T}S_{i}W_{a}) \right]$ | (S23) |
| --- | --- |

where $P_{i}$ is the *a prosteriori* probability of *x* belonging to class *i*. It is determined by the ratio between the number of measurements corresponding to a category and the total number of measurements corresponding to all categories.

The flow diagram of the FDA algorithm is given in Fig. S2.


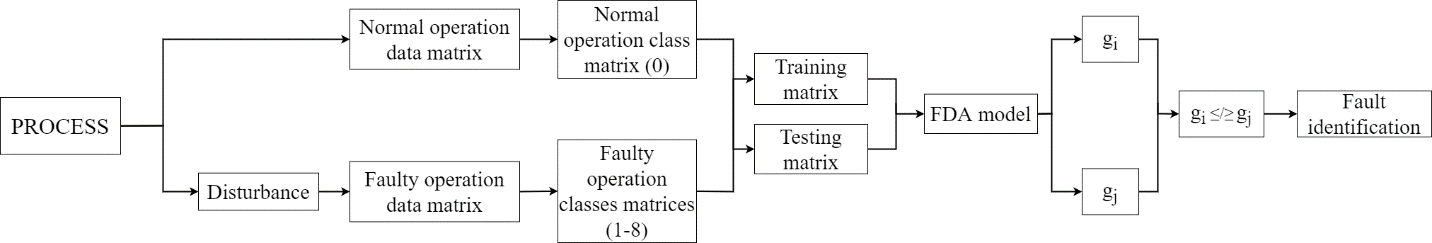


**Fig. S2** Flow diagram of the FDA algorithm

**Results and discussion**

PCA model construction and detection results

Table S4. Cumulative percent of variance and threshold values for the different number of considered principal components

| Number of selected principal components (*k*) | CPV*_k_* (%) | $T_{\alpha}^{2}$ | SPE_α_ |
| --- | --- | --- | --- |
| 7 | 98.11 | 18.54 | 1.62 |
| 8 | 98.98 | 20.16 | 0.97 |
| 9 | 99.52 | 21.75 | 0.38 |

The scree plot in Fig. S3 shows that 8 eigenvalues are sufficient and adequately capture the process variability. The covariance matrix C and the diagonal matrix S were computed based on this selection.

**Fig. S3** Scree plot showing the model eigenvalues

Fault detection results

First, the proposed PCA model-based detection methodology was applied to the training (regular, fault free) data set. As shown in Fig. S4, the T^2^ and SPE values from this data set, presented against their thresholds, confirm the fault-free scenario. SPE index performs a perfect detection, while T^2^ index is also good, with exception of a very limited set of points that belong to the days no. 105, 119 and 133. For measurements taken in those days extreme values occurred for the following process variables: minimum values for *S_O_* (settler bottom), *Q_NR_* and maximum for *S_NO_*, *S_NH_* (settler bottom) and for *S_NO_* (effluent). These results demonstrate that the SPE statistics provides reliable outcomes for assessing the normal operation mode. Changes in the interactions between variables, such as those specific to the sensor faults, are well known to be efficiently detected with the SPE method, whereas variations in operating conditions are typically and mostly identified with the T^2^ method (Yoo et al., 2004). This means that the SPE statistics provide the most relevant data in normal operation mode.


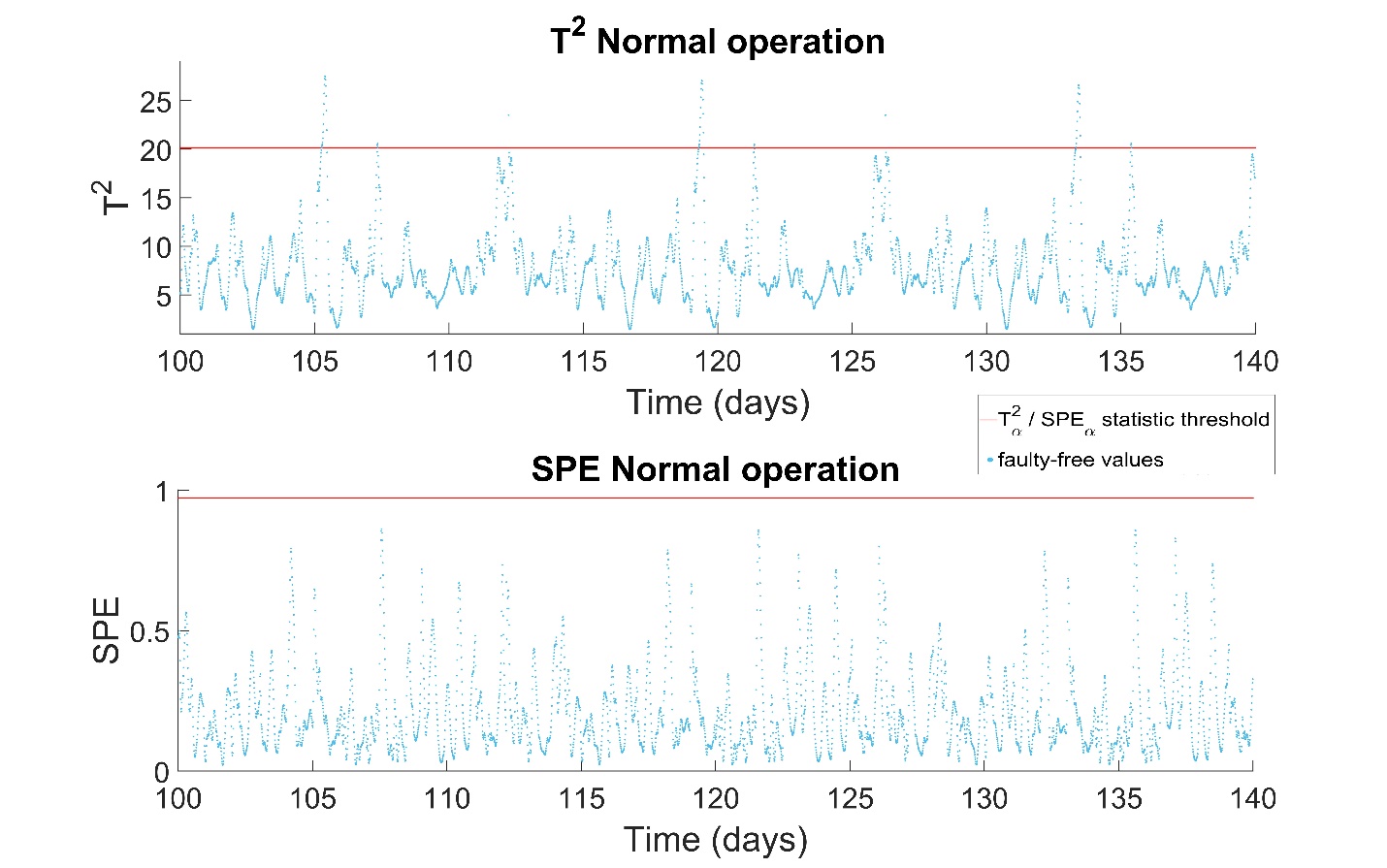


**Fig. S4** T^2^ and SPE graphical representations for the fault-free operation

As it may be observed from Fig. S5 the constant additive error was detected after 1.5h from its starting action. The SPE plot shows a better detection of the faulty regime than the T^2^ one, excluding a limited set of measurement from the days no. 145, 152 and 159.


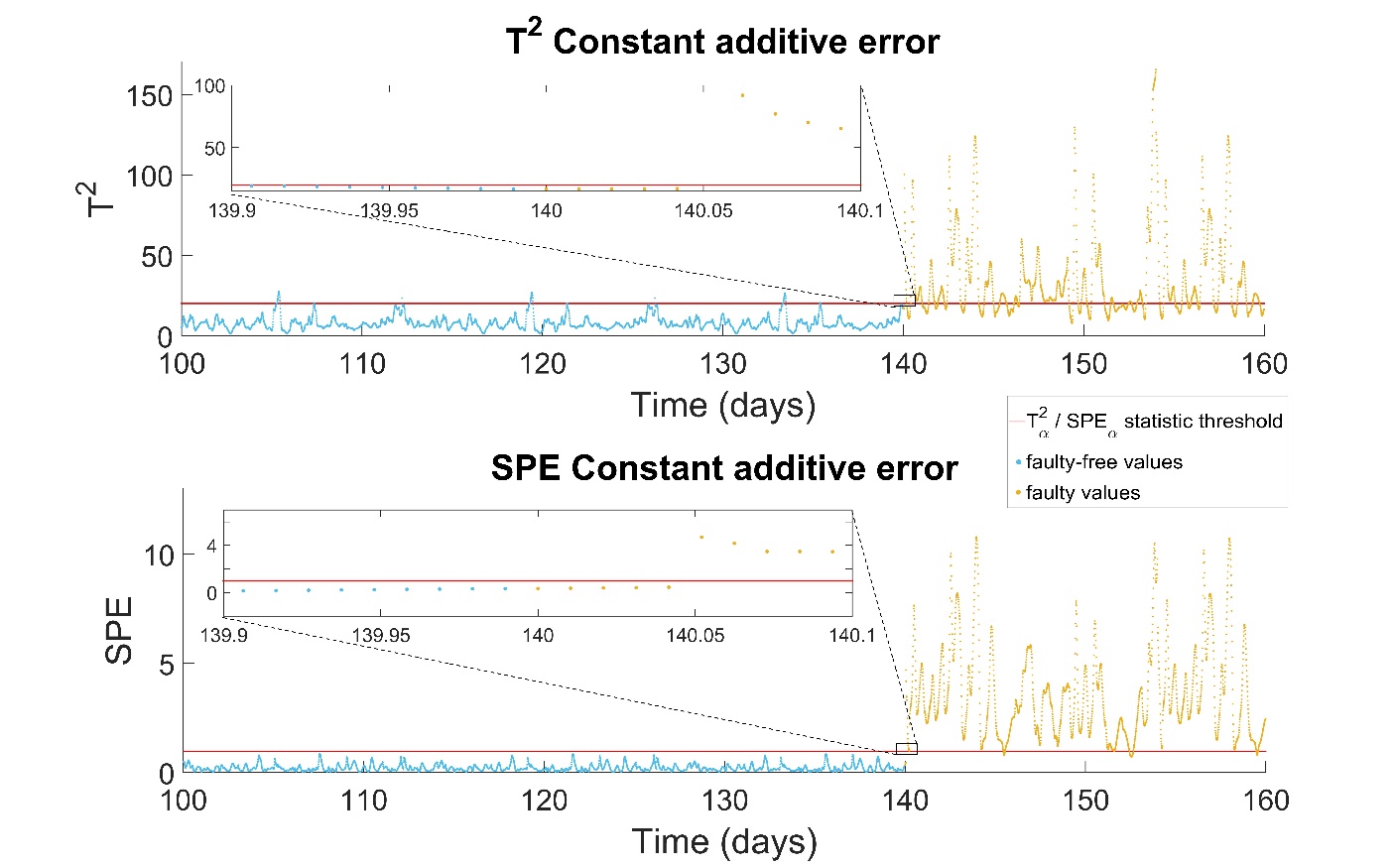


**Fig. S5** T^2^ and SPE graphical representations for the constant additive error

SPE method proved to detect the ramp changing error in time faster than T^2^, in 19.25h. The NO sensor fault detection with T^2^ metrics took 35.25h, as can be seen in Fig. S6. This error was expected to have an extended detection time because the faulty signal grows slowly over time and only when its amplitude is large enough it will exceed the statistical threshold.


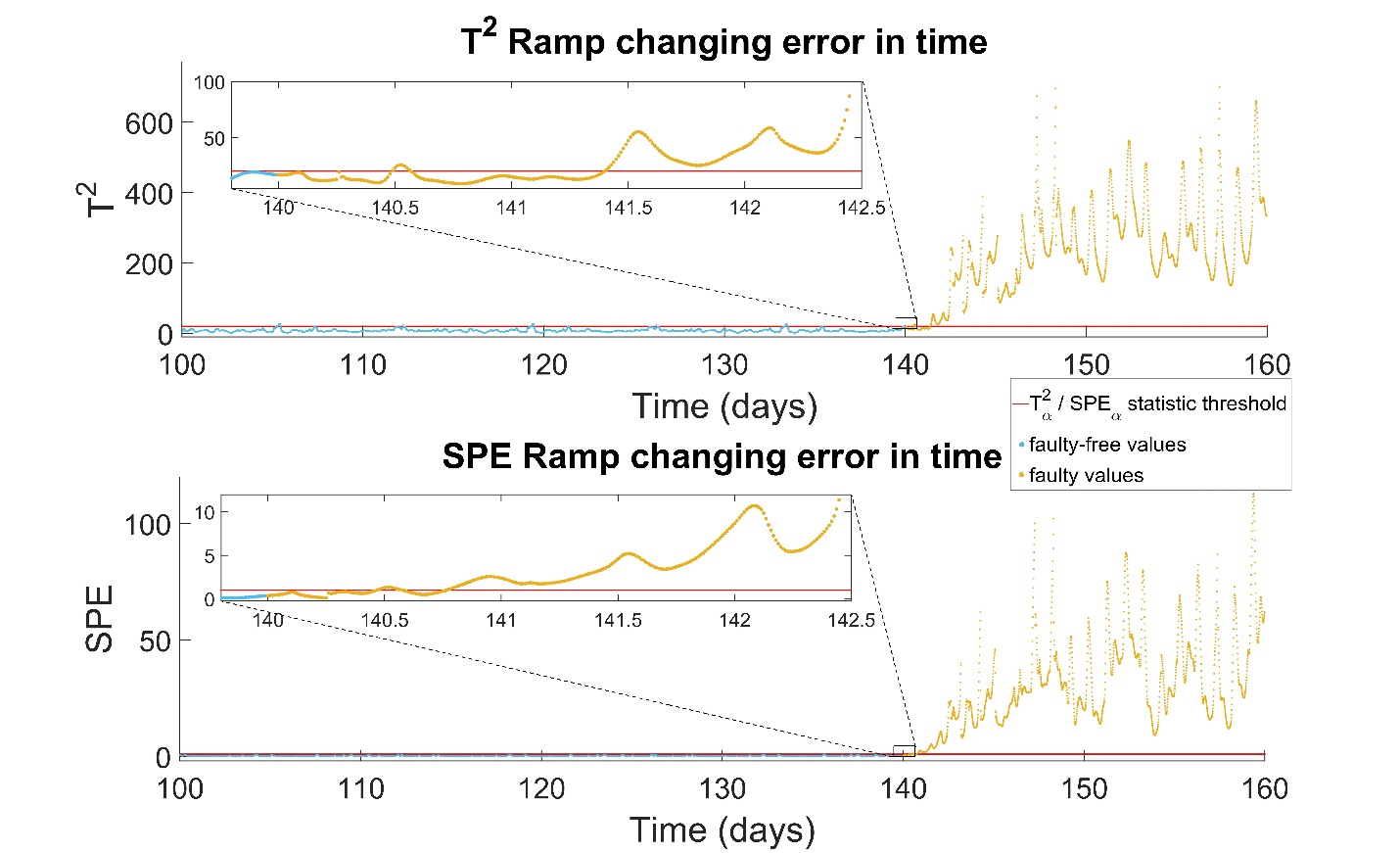


**Fig. S6** T^2^ and SPE graphical representations for the ramp changing error in time

Fig. S7 presents the incorrect amplification fault detection. T^2^ method initially detects the fault in just 1.5h and confirms the fault presence for the whole period after 6.5h time moment, while the SPE method recognises the fault after 2.75h.


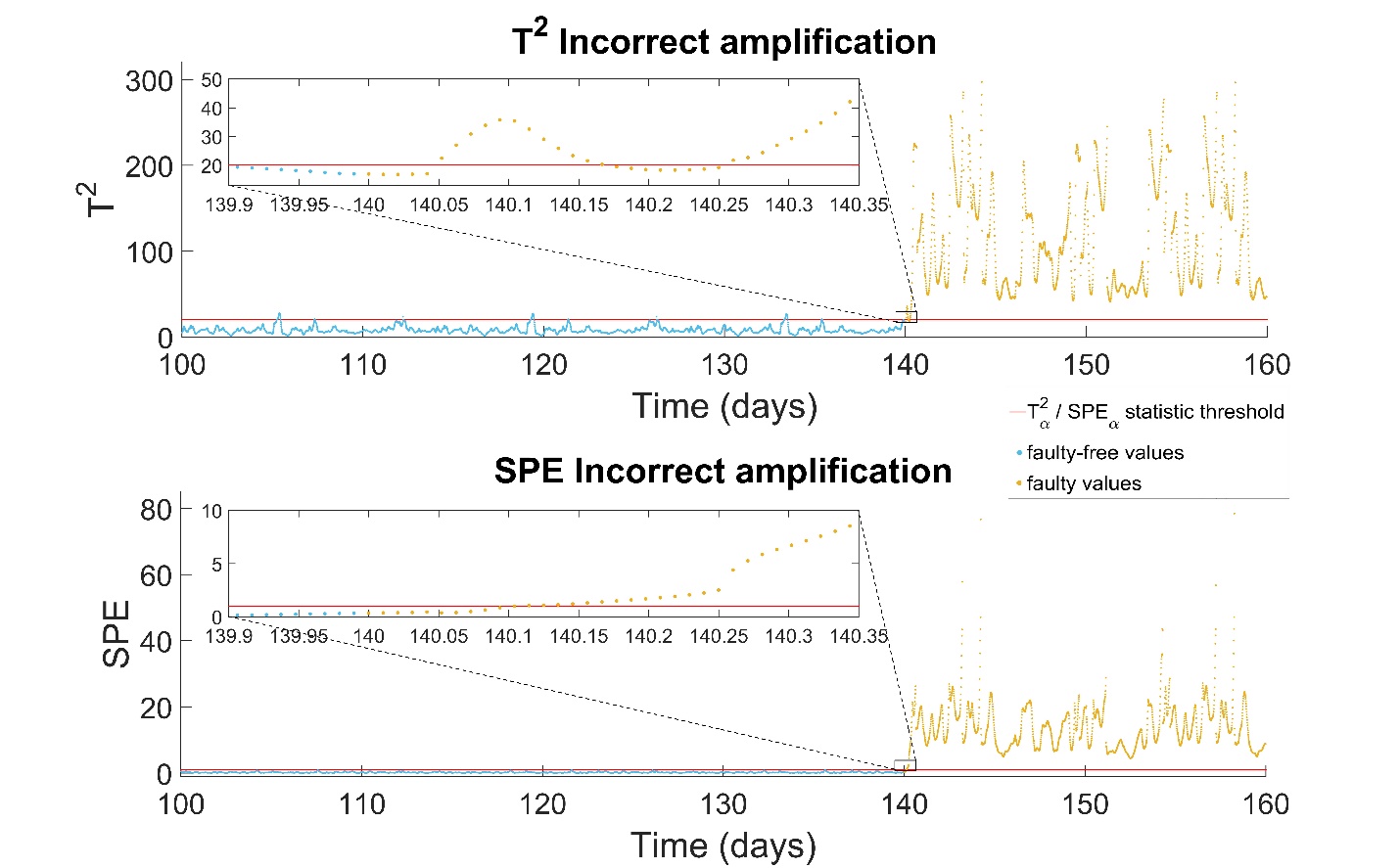


**Fig. S7** T^2^ and SPE graphical representations for the incorrect amplification error

T^2^ metric detects the random additive error in 1.75h and SPE confirms its presence after 3.75h., as shown in Fig. S8. Following this time instances there are few values associated to exceptions of the correct fault detection.

**
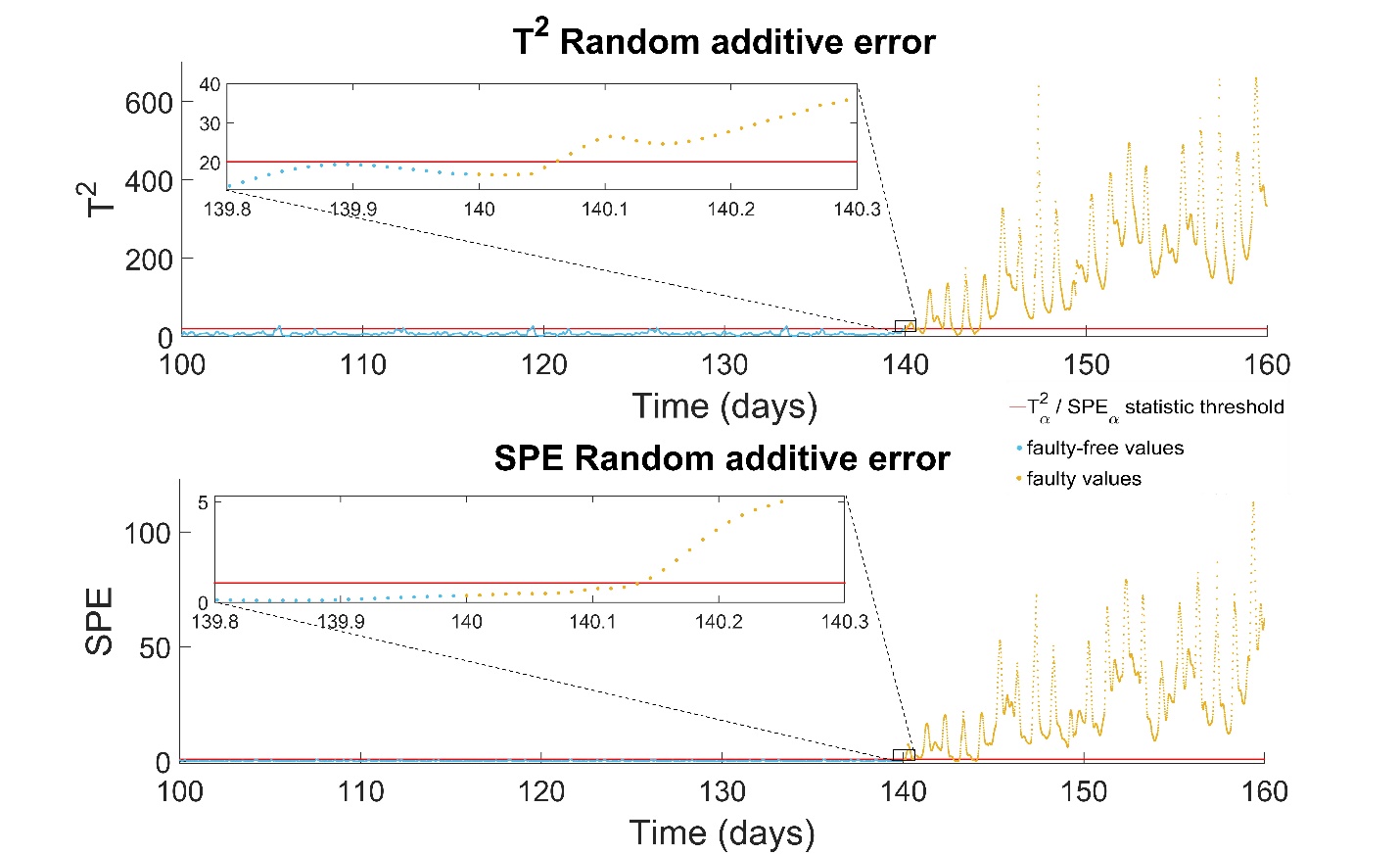
**

**Fig. S8** T^2^ and SPE graphical representations for the random additive error

Fig. S9 displays the detection of the unchanging sensor value error. In this case both methods proved to be equally effective and fast, detecting the fault in 1.5h.


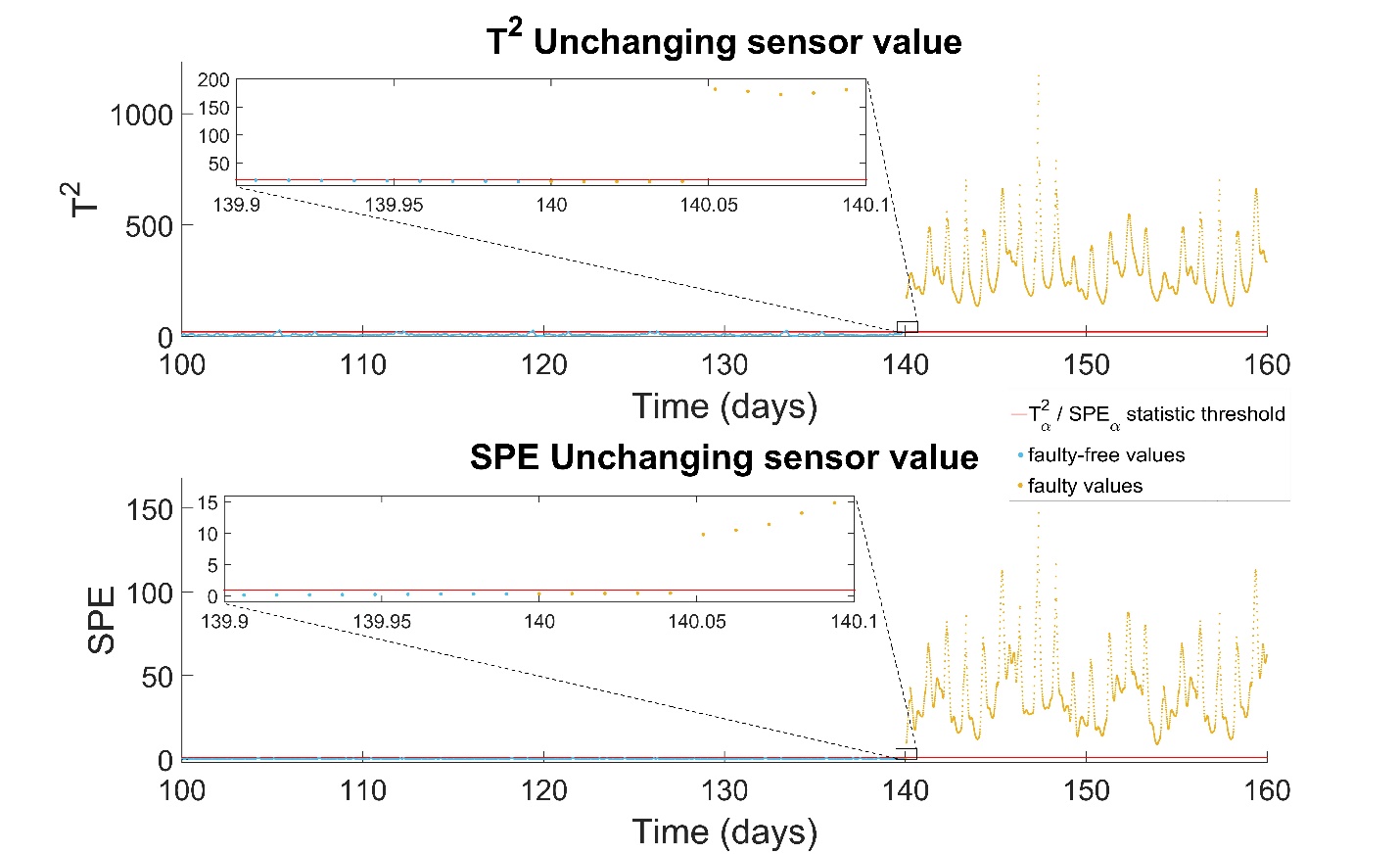


**Fig. S9** T^2^ and SPE graphical representations for the unchanging sensor value

Energy costs assessment for faulty operation

Table S5 shows a comparison of energy prices based on the source or technology used to produce it.

Table S5. Costs of different energy sources

| Source/Technology | €cents/kWh |
| --- | --- |
| Lignite | 9.12 |
| Coal | 8.80 |
| Gas (CCGT^a^) | 6.81 |
| Nuclear | 6.62 |
| Wind onshore | 4.80 |
| Wind offshore | 8.45 |
| Solar PV^b^ commercial | 5.38 |
| Solar PV residential | 12.10 |
| Solar thermal (CSP^c^) | 11.62 |
| Hydro reservoir | 6.91 |
| Hydro run of river | 6.53 |
| Geothermal | 9.50 |
| Biomass | 11.33 |

^a^ CCGT - combined cycle gas turbines

^b^ PV - photovoltaic

^c^ CSP - concentrating solar power

Table S5. Costs of different energy sources

The sum of the AE and PE energy costs computed for regular and error affected operation is source dependent as can be seen in Table S6.

Table S6. Source depending sum of AE and PE energy costs computed for regular and error affected operation

| Source/Technology | Daily operation costs (€) | | | | | |
| --- | --- | --- | --- | --- | --- | --- |
|  | Normal operation | Constant additive error | Ramp changing error in time | Incorrect amplification error | Random additive error | Unchanging sensor value error |
| Lignite | 1,671 | 1,722 | 1,833 | 1,774 | 1,823 | 1,848 |
| Coal | 1,612 | 1,662 | 1,768 | 1,712 | 1,759 | 1,783 |
| CCGT | 1,249 | 1,287 | 1,370 | 1,326 | 1,362 | 1,381 |
| Nuclear | 1,214 | 1,251 | 1,331 | 1,289 | 1,324 | 1,342 |
| Onshore wind | 879 | 907 | 965 | 934 | 959 | 973 |
| Offshore wind | 1,548 | 1,596 | 1,698 | 1,644 | 1,688 | 1,712 |
| Solar PV commercial | 985 | 1,015 | 1,080 | 1,046 | 1,074 | 1,089 |
| Solar PV residential | 2,216 | 2,284 | 2,431 | 2,353 | 2,417 | 2,451 |
| Solar thermal (CSP) | 2,128 | 2,194 | 2,334 | 2,260 | 2,321 | 2,354 |
| Hydro reservoir | 1,266 | 1,305 | 1,389 | 1,345 | 1,381 | 1,401 |
| Hydro run of river | 1,196 | 1,233 | 1,312 | 1,270 | 1,305 | 1,323 |
| Geothermal | 1,741 | 1,795 | 1,910 | 1,849 | 1,899 | 1,926 |
| Biomass | 2,075 | 2,139 | 2,276 | 2,204 | 2,264 | 2,295 |
